# LCK‐Mediated RIPK3 Activation Controls Double‐Positive Thymocyte Proliferation and Restrains Thymic Lymphoma by Regulating the PP2A‐ERK Axis

**DOI:** 10.1002/advs.202204522

**Published:** 2022-09-25

**Authors:** Sung‐Min Hwang, Yu‐Jin Ha, Gi‐Bang Koo, Hyun‐Jin Noh, A‐Yeon Lee, Byeong‐Ju Kim, Sun Mi Hong, Michael J. Morgan, Seong‐il Eyun, Dakeun Lee, Jae‐Seok Roe, Youngsoo Lee, You‐Sun Kim

**Affiliations:** ^1^ Department of Biochemistry and Molecular Biology Ajou University School of Medicine 164 Worldcup‐ro, Yeongtong‐gu, Suwon Gyeonggi‐do 16499 Republic of Korea; ^2^ Sandra and Edward Meyer Cancer Center and Department of Obstetrics and Gynecology Weill Cornell Medicine New York NY 10065 USA; ^3^ Department of Biomedical Sciences Graduate School of Ajou University 164 Worldcup‐ro, Yeongtong‐gu, Suwon Gyeonggi‐do 16499 Republic of Korea; ^4^ Department of Natural Sciences Northeastern State University Tahlequah OK 74464 USA; ^5^ Department of Life Science Chung‐Ang University Seoul 06973 Republic of Korea; ^6^ Department of Pathology Ajou University School of Medicine 164 Worldcup‐ro, Yeongtong‐gu, Suwon Gyeonggi‐do 16499 Republic of Korea; ^7^ Department of Biochemistry College of Life Science and Biotechnology Yonsei University Seoul 03722 Republic of Korea; ^8^ Institute of Medical Science Ajou University School of Medicine 164 Worldcup‐ro, Yeongtong‐gu, Suwon Gyeonggi‐do 16499 Republic of Korea

**Keywords:** double‐positive thymocytes, extracellular signal‐regulated kinase (ERK), lymphocyte‐specific protein tyrosine kinase (LCK), protein phosphatase 2A (PP2A), receptor‐interacting protein kinase 3 (RIPK3), thymic lymphoma

## Abstract

Receptor‐interacting protein kinase 3 (RIPK3) is the primary regulator of necroptotic cell death. RIPK3 expression is often silenced in various cancer cells, which suggests that it may have tumor suppressor properties. However, the exact mechanism by which RIPK3 negatively regulates cancer development and progression remains unclear. This report indicates that RIPK3 acts as a potent regulator of the homeostatic proliferation of CD4^+^CD8^+^ double‐positive (DP) thymocytes. Abnormal proliferation of RIPK3‐deficient DP thymocytes occurs independently of the well‐known role for RIPK3 in necroptosis (upstream of MLKL activation), and is associated with an incidental thymic mass, likely thymic hyperplasia. In addition, *Ripk3*‐null mice develop increased thymic tumor formation accompanied by reduced host survival in the context of an *N*‐ethyl‐*N*‐nitrosourea (ENU)‐induced tumor model. Moreover, RIPK3 deficiency in *p53*‐null mice promotes thymic lymphoma development via upregulated extracellular signal‐regulated kinase (ERK) signaling, which correlates with markedly reduced survival rates. Mechanistically, lymphocyte‐specific protein tyrosine kinase (LCK) activates RIPK3, which in turn leads to increases in the phosphatase activity of protein phosphatase 2 (PP2A), thereby suppressing hyper‐activation of ERK in DP thymocytes. Overall, these findings suggest that a RIPK3‐PP2A‐ERK signaling axis regulates DP thymocyte homeostasis and may provide a potential therapeutic target to improve thymic lymphoma therapies.

## Graphical abstract

The thymus is a specialized microenvironment for T lymphocyte development and maturation. Loss of receptor‐interacting protein kinase 3 (RIPK3), an essential protein in necroptosis, causes hyper‐proliferation of CD4^+^CD8^+^ double‐positive (DP) thymocytes independently of its well‐known necroptotic role. This RIPK3‐mediated regulatory axis serves in maintaining the homeostasis of the DP thymocytes population and can provide a potential therapeutic target in cancer patients.

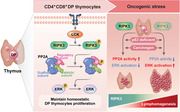

## 1 Introduction

Receptor‐interacting protein kinase 3 (RIPK3) is a serine/threonine‐protein kinase that induces a form of programmed cell death called necroptosis.^[^ ^1^ ^]^ Multiple ligand‐dependent cell surface receptor signaling pathways, such as tumor necrosis factor (TNF) superfamily death receptors, interferon receptors (IFNRs), and toll‐like receptors (TLRs), can lead to phosphorylation of RIPK3, typically within a necrotic signaling complex formed in combination with RIPK1 or other RIP‐homotypic‐interaction‐motif (RHIM) domain‐containing proteins. RIPK3 then phosphorylates and activates the pore‐forming mixed‐lineage kinase domain‐like pseudokinase (MLKL), which is responsible for plasma membrane permeability and cell death.^[^ ^2^ ^]^ Consequently, the RIPK3‐mediated necroptosis pathway often induces the release of pro‐inflammatory cytokines and damage‐associated molecular patterns (DAMPs), triggering an inflammatory immune response.^[^ ^3^ ^]^

Recently, it has been demonstrated that RIPK3 has several different roles in the cell, and perhaps more than one function within necroptosis itself, which in some cases contribute to various immune diseases.^[^ ^4^ ^]^ In support of this, we recently reported that RIPK3 promotes the transcription of catabolic factors, such as MMP3, MMP13, COX2, and ADAMTS4, which aggravate the pathogenesis of osteoarthritis.^[^ ^5^ ^]^ In addition, RIPK3 signaling is indispensable for limiting several infectious and inflammatory conditions, including Zika virus (ZIKV) infection, cowpox virus (CPXV) infection,^[^ ^6^ ^]^ neuroinflammation, ^[^ ^7^ ^]^ and toll‐like receptor 4 (TLR 4)‐mediated inflammation.^[^ ^8^ ^]^ These results support the notion that RIPK3 is involved in inflammation‐mediated pathogenesis in a cell death‐specific manner. On the other hand, many studies have shown that RIPK3 expression in tumor tissues is highly correlated with patient survival.^[^ ^3^, ^9^ ^]^ Along these lines, we have previously shown that DNA methylation significantly silences RIPK3 expression in many cancer cells and impairs anti‐tumorigenic processes.^[^ ^9a^ ^]^ RIPK3 can suppress myeloid transformation by promoting cell death and differentiation of leukemia initiating cells.^[^ ^10^ ^]^ Conversely, RIPK3 plays a role in promoting pancreatic oncogenesis by mediating CXCL1 and Mincle‐induced immune suppression by inducing necroptosis in pancreatic ductal epithelium,^[^ ^11^ ^]^ suggesting that there might be the context‐dependent roles of RIPK3‐mediated necroptotic cell death. More recently, RIPK3 was shown to contribute immunogenic cell death that leads to anti‐tumor immune responses through promoting cross‐priming of CD8^+^ T cell vaccination responses in tumor microenvironment.^[^ ^12^ ^]^ Therefore, understanding the mechanisms by which RIPK3 contributes to the anti‐tumor response could be a promising target for immunotherapy in many cancer diseases.

The thymus is a specialized microenvironment that is indispensable for T lymphocyte development and maturation.^[^ ^13^ ^]^ Thymocytes interact with local stromal cells and antigen‐presenting cells (APCs), undergoing several distinct steps that are regulated by soluble growth and survival factors, allowing immature CD4^+^CD8^+^ double‐positive (DP) thymocytes to develop and mature into CD4^+^ or CD8^+^ T lymphocytes.^[^ ^14^ ^]^ The maintenance of a well‐controlled T lymphocyte population is also quite important for thymic homeostasis. In this respect, another form of programmed cell death, apoptosis, plays a crucial role in eliminating non‐functional or autoreactive T lymphocytes during various transitional stages in the thymus. Necroptosis has also been shown to contribute to the maintenance of T cell homeostasis.^[^ ^15^ ^]^ For example, the elimination of excessive and abnormally proliferating T lymphocytes in the absence of caspase‐8 occurs via necroptosis.^[^ ^16^ ^]^ Along these lines, it has been reported that abnormal proliferation of cells present in the thymus leads to lymphoblastic thymic lymphoma.^[^ ^17^ ^]^ This suggests the possibility that a deficiency in the RIPK3‐mediated necroptosis pathway may contribute to the pathogenesis of thymic lymphoma.

This study demonstrates that RIPK3 acts as a negative regulator of thymic lymphoma initiation and progression by inhibiting the proliferation of abnormal CD4^+^CD8^+^ DP thymocytes. Abnormal proliferation of RIPK3‐deficient DP T cells is not related to the lack of a functional necroptosis signaling pathway. Genetic deletion of *Ripk3* in mice results in a spontaneously high incidence of thymic lymphoma along with a shortened overall host survival. Mechanistically, TCR‐mediated lymphocyte‐specific protein tyrosine kinase (LCK) activates RIPK3 to increase the phosphatase activity of protein phosphatase 2 (PP2A), preventing overactivation of extracellular signal‐regulated kinase (ERK) in DP thymocytes and limiting the progression of thymic lymphoma. Hence, this study suggests that the RIPK3‐PP2A‐ERK axis can serve as a new axis for maintaining the homeostasis of the DP thymocytes population and further regulating thymic lymphoma development and progression.

## 2 Results

### 2.1 RIPK3 Deficiency Leads to Hyperproliferation of DP Thymocytes in the Thymus

**Figure 1:**
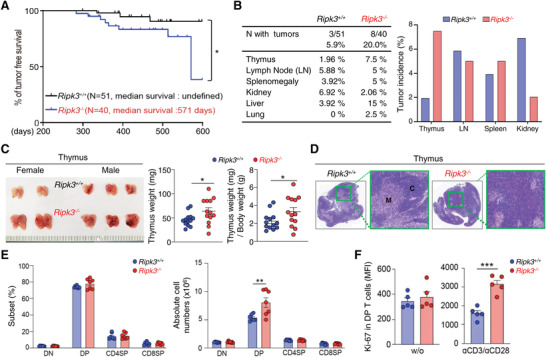
Genetic loss of RIPK3 leads to hyperproliferation of DP thymocytes contributing to thymic lymphoma. A) Kaplan–Meier survival curve (Long‐rank test) in *Ripk3^+/+^* (*n* = 51) and *Ripk3^−/−^* (*n* = 40) mice. B) Spontaneous tumors found in various organs and their frequency in *Ripk3^+/+^* and *Ripk3^−/−^* animals. C) Thymus from *Ripk3^+/+^* and *Ripk3^−/−^* was shown (left panel) and its weight was shown in the graph (right panel). (*n* = 13 for each group). D) Representative H&E images of thymus from both *Ripk3^+/+^* and *Ripk3^−/−^* mice. E) Percentage of the thymic T cell subsets (left panel) and the absolute cell number (right panel) by the indicated thymic subsets are shown in each graph. (*n* = 7 for each group). F) Relative fluorescence of Ki‐67 in CD4^+^CD8^+^ double positive (DP) T cells under anti‐CD3/CD28 stimulation. Total thymocytes from *Ripk3^+/+^* (*n* = 5) and *Ripk3^−/−^* (*n* = 5) mice were stimulated with anti‐CD3 (1 µg mL^−1^) and anti‐CD28 (2 µg mL^−1^) or without, and then Ki‐67 in DP cell was measured by FACS. Statistical analyses were performed using the two‐tailed unpaired Student *t*‐test. P values below 0.05 were considered significant in the following manner: *p < 0.05, **p < 0.01, ***p < 0.001.

Extensive loss/reduction of RIPK3 expression in many cancers suggests its loss provides some kind of selective advantage to tumor cells,^[^ ^9a^ ^]^ while previous characterization of RIPK3 knockout mice did not observe spontaneous tumor formation in these studies.^[^ ^18^ ^]^ We therefore sought to define the role of RIPK3 in tumor suppression by using *Ripk3* ^−/−^ mice where the tumor‐promoting/enhancing conditions might be more readily seen, such as in carcinogen‐induced models or in models where other tumor suppressors were lost. Initially, however, we looked solely at the effect of RIPK3 deletion in mice with mixed genetic background of B6 and 129/Sv [B6/129], since it is well known that animals in the B6 genetic background have a lower susceptibility to tumors compared to 129 mice,^[^ ^19^ ^]^ and previous studies examined RIPK3 loss exclusively maintained in the C57BL/6 (B6) genetic background. Furthermore, we noted that in previous descriptions of the *Ripk3* ^−/−^ mouse phenotype, the shorter‐term periods over that the mice were observed might not have been sufficient to fully evaluate spontaneous tumorigenesis under RIPK3‐deficient conditions. Thus, we followed spontaneous tumorigenesis in *Ripk3* ^+/+^ and *Ripk3* ^−/−^ mice in the mixed B6/129 background over their entire life spans (Figure S1A, Supporting Information) with appropriate siblings as control groups from the breeding in a mixed background, so that the genetic variation in control groups matched the variation in *Rikp3* ^−/−^ mice. We first confirmed that germline *Ripk3* ^−/−^ mice in our animal facility maintained under specific‐pathogen‐free conditions were free of any signs or symptoms associated with particular disease phenotypes as previously reported.^[^ ^18a^ ^]^ Then we observed that over full mice lifetimes, RIPK3‐deficient mice showed a reduced survival rate accompanied by a higher incidence of spontaneous tumor formation in various tissues, including the liver and lungs (**Figure** **1**A). Notably, the incidence of tumors in the thymus was significantly higher than that in other tissues (Figure 1B). Indeed, upon close inspection, we observed that 5 to 7‐week‐old *Ripk3* ^−/−^ mice developed an early stage of thymic hyperplasia, characterized by an increase in the size and weight of the thymus (Figure 1C; Figure S1B, Supporting Information) accompanied by abnormal thymic architecture (Figure 1D). These results raised the possibility that RIPK3 loss sensitized mice to tumorigenesis within the thymus, perhaps by RIPK3‐deficient thymus‐derived cells. To dissect which type of RIPK3‐deficient cells may be promoting hyperplasia, we compared the different thymus‐derived cell populations in *Ripk3* ^+/+^ and *Ripk3* ^−/‐^ mice. We found that RIPK3 deficiency did not alter CD11c^+^ and F4/80^+^ populations (Figure S1C, Supporting Information), but interestingly increased the number of CD4^+^CD8^+^ double positive (DP) thymocytes in the thymus (Figure 1E). RIPK3‐deficient DP thymocytes showed increased proliferation, which was further significantly increased upon anti‐CD3/28 stimulation in vitro (Figure 1F), suggesting that RIPK3 may be involved in T cell receptor (TCR) signaling‐mediated DP thymocytes proliferation.

We further examined whether RIPK3 deficiency would affect the proportion of various immune cells in the periphery. Of note, there was no significant difference in the size, weight, and architecture of the spleen of *Ripk3* ^−/‐^ mice (Figure S1D,E, Supporting Information). In addition, no difference in CD4 and CD8 T cell populations was observed in the spleen in *Ripk3* ^+/+^ and *Ripk3* ^−/‐^ mice (Figure S1F, Supporting Information), nor were differences observed in naïve (CD62^hi^CD44^lo^) and effector T cell (CD62^lo^CD44^hi^) populations (Figure S1G, Supporting Information). Furthermore, there were no abnormalities in the populations of CD11c^−^F4/80^lo^, CD11c^−^F4/80^hi^, and CD11c^+^F4/80^−^cells representing myeloid cell populations in the RIPK3‐deficient spleen. (Figure S1H, Supporting Information). Taken together, these findings suggest that loss of RIPK3 is responsible for the hyperproliferation of DP thymocytes that initiate the early stage of thymic tumorigenesis.

### 2.2 Increased RIPK3‐Deficient DP Thymocyte Proliferation is Independent of the Canonical RIPK3‐MLKL Necroptosis Pathway

**Figure 2:**
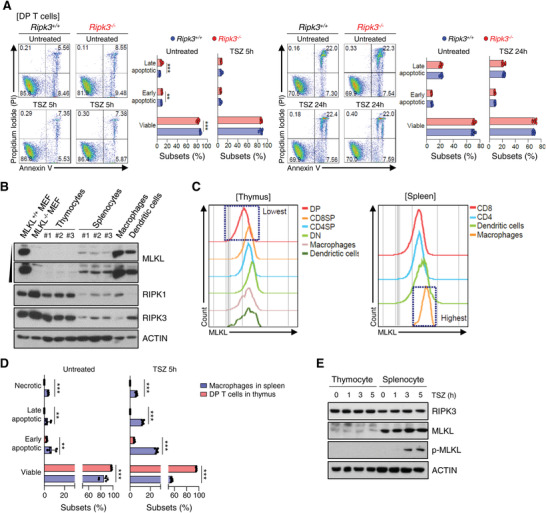
RIPK3‐MLKL axis does not impact the proliferation and death of DP thymocytes. A) Total thymocytes from *Ripk3^+/+^* (*n* = 6) and *Ripk3^−/−^* (*n* = 6) mice were treated with TNF‐*α* (50 ng mL^−1^), z‐VAD (20 µm) and Smac mimetic (200 nm) for 5 and 24 h. Dead cells were identified by Annexin V and PI staining. B) The expression level of MLKL protein in primary immune cells. MLKL expression was measured by Western blot analysis. C) Representative histograms were the MLKL expression of the indicated thymic and splenic subsets. Expression levels of MLKL were measured by flow cytometry. Total thymocytes and splenocytes were treated with TNF‐*α* (50 ng mL^−1^), z‐VAD (20 µm) and Smac mimetic (200 nm) for the indicated time or without, and cells were analyzed by Annexin V and Propidium Iodide (PI) staining and measured by D) Flow Cytometry. RIPK3, MLKL and *p*‐MLKL protein was measured by E) Western blot analysis. Statistical analyses were performed using the two‐tailed unpaired Student *t*‐test. P values below 0.05 were considered significant in the following manner: *p < 0.05, **p < 0.01, ***p < 0.001.

Since RIPK3 is the major regulator of necroptosis‐induced cell death, we speculated that RIPK3‐deficient DP thymocytes would not undergo necroptosis, resulting in abnormal proliferation in the thymus. To prove this notion, *Ripk3* ^+/+^ and *Ripk3* ^−/‐^ DP thymocytes were cultured in a medium containing the combination of tumor necrosis factor alpha (T), a Smac mimetic (S) and the caspase inhibitor zVAD (Z), (described as TSZ), that induces cell necroptosis in vitro. Contrary to our expectations, there were no significant differences in cell viability between *Ripk3* ^+/+^ and *Ripk3* ^−/‐^ DP thymocytes under TSZ‐treated conditions, albeit a slight difference at the early time point without TSZ treatment (**Figure** **2**A; Figure S2A, Supporting Information). These results suggest the possibility that the increased DP thymocyte population could be independent of the canonical RIPK3‐mediated necroptosis. Therefore, we hypothesized that there would be differences in the expression of necroptosis players, such as RIPK1, 3 and MLKL in DP thymocytes, unlike cells undergoing necroptosis. By employing Gene Atlas analyses, we found that RIPK1 and RIPK3 expression were similar in DP thymocytes and various other immune cells (Figure S2B,C, Supporting Information). MLKL is expressed at particularly low levels in many immune cells, including DP thymocytes, whereas it is expressed at very high levels in macrophages (Figure 2B,C; Figure S2D, Supporting Information), suggesting that the canonical necroptosis pathway may not be involved in DP thymocytes due to very low expression of MLKL. Indeed, in vitro analysis revealed that DP thymocytes underwent significantly less cell death than macrophages under TSZ conditions (Figure 2D). In parallel with these findings, the level of p‐MLKL, which indicates the activation status of MLKL induced by RIPK3, was also increased in splenic macrophages (Figure 2E).

Additionally, we directly examined whether the expression of MLKL was reduced in thymic lymphoma cells. For this purpose, we used the thymic T cell‐derived lymphoma cell line (L5178) and T cell‐derived leukemia cell lines (EL‐4 and L1210). Of note, we added the macrophage‐derived RAW 264.7 cell line as a positive control for MLKL expression. RIPK1 and RIPK3 were expressed in all cell lines, but MLKL was barely expressed in the L5178 cell line when examined via western blot (Figure S2E, Supporting Information) and flow cytometry (Figure S2F, Supporting Information) analysis, respectively. Taken together, this suggests a unique function of RIPK3 in regulating DP thymocytes population independent of the canonical RIPK3‐MLKL necroptosis pathway in the thymus.

### 2.3 RIPK3 Deficiency Accelerates Tumor Progression in a Carcinogen‐Induced Mouse Model

**Figure 3:**
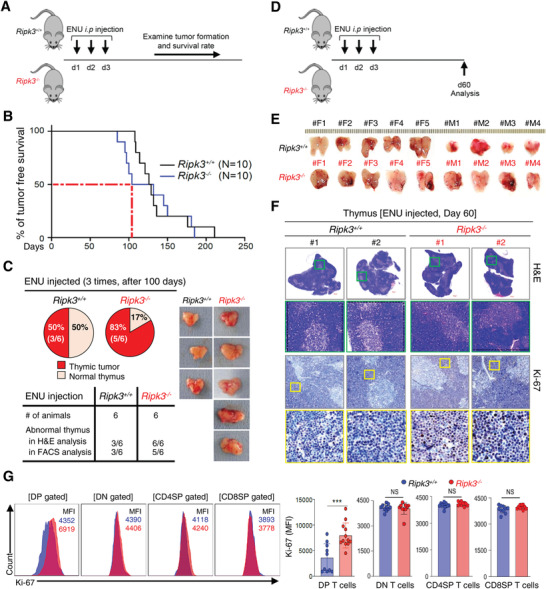
Carcinogen‐induced thymic tumorigenesis was accelerated in RIPK3‐deficient mice. A) Intraperitoneal injection of ENU (*N*‐ethyl‐*N*‐nitrosourea) at around postnatal day 13 ∼15 (three‐time; 13∼15 day) was performed to both *Ripk3^+/+^* and *Ripk3^−/−^* mice. B) Kaplan–Meier survival curve (Long‐rank test) after 3 consecutive daily ENU injections into *Ripk3* wildtype and *Ripk3* knockout mice (*n* = 10 for each group). Around 105 days after ENU injection, 50% of *Ripk3^−/−^* mice succumbed to tumors (red dotted line). C) All of animals were examined at 100 days after 3 consecutive daily ENU injection to *Ripk3^+/+^* (*n* = 6) and *Ripk3^−/−^* (*n* = 6) mice. The summary of tumor incidence in this ENU injection experiment. 83% of the *Ripk3^−/‐^* animals developed thymic lymphoma at the microscopic level. D) Schematic diagram of an ENU injection experiment. E) Gross view of the abnormally big thymus removed from ENU injected *Ripk3^+/+^* and *Ripk3^−/−^* mice at day 60. *Ripk3* ^−/−^ thymus showed a more hyperplasia than *Ripk3^+/+^* thymus. F) Representative images of hematoxylin and eosin (H&E) stained sections and Ki‐67 staining of thymus from both ENU injected *Ripk3^+/+^* and *Ripk3^−/−^* mice. G) Relative fluorescence of Ki‐67 in indicated thymic T cells (blue box in *Ripk3^+/+^* and red box in *Ripk3^−/−^*) analyzed by Flow Cytometry (*n* = 10 ∼ 12 for each group). Statistical analyses were performed using the two‐tailed unpaired Student *t*‐test or log rank test. P values below 0.05 were considered significant in the following manner: *p < 0.05, **p < 0.01, ***p < 0.001.

Since we observed that tumor formation spontaneously increased in an age‐dependent manner in *Ripk3* ^−/‐^ mice, we wished to examine whether RIPK3 deficiency promotes susceptibility to tumor progression triggered by carcinogens at a relatively young age. For this purpose, *N*‐ethyl‐*N*‐nitrosourea (ENU), a potent mutagen, was sequentially injected for three times to evaluate rapid tumor development between two groups (*Ripk3^+/+^* versus *Ripk3^−/−^* mice group) (**Figure** **3**A). As expected, *Ripk3* ^−/‐^ mice showed a reduced median survival compared to *Ripk3^+/+^* mice under ENU‐treated conditions (Figure 3B). Upon further examination, 100 days after ENU injection, 83% of the *Ripk3^−/‐^* mice had an abnormal thymus, compared to 50% of *Ripk3^+/+^* mice, thus reflecting the phenotype of early thymic lymphoma (as observed by the subpopulation of CD4 and CD8 by flow cytometry analysis, Architecture of medullar and cortex by H&E staining analysis) (Figure 3C). These results suggest that RIPK3 deficiency can expedite tumorigenesis at a relatively young age after ENU injection.

To prove this notion, we further investigated the thymic phenotypes, as well as thymocytes themselves, in *Ripk3* ^+/+^ and *Ripk3^−/−^* mice 60 days after ENU injection (Figure 3D). Indeed, *Ripk3* ^−/−^ mice showed a greater proportion of thymic hyperplasia than *Ripk3^+/+^* mice, characterized by an increase in the size/weight of the thymus (Figure 3E; Figure S3A, Supporting Information). Moreover, more Ki‐67^+^ cells were observed in RIPK3‐deficient thymi with a higher rate of abnormal thymus architecture than in RIPK3‐present thymi (Figure 3F). Importantly, Ki‐67 expression was significantly increased only in DP thymocytes (Figure 3G), supporting the observations made previously regarding the increase of *Ripk3^−/−^* DP thymocytes (Figure 1E). However, there were no significant phenotypic differences or matured CD4 and CD8 T cell populations in other lymphoid organs, such as the spleen, peripheral lymph node (pLN) and mesenteric lymph node (mLN) when comparing *Ripk3* ^+/+^ and *Ripk3^−/−^* mice (Figure S3B–E, Supporting Information). In addition, Ki‐67 expression was not different in CD4 and CD8 T cell populations in spleen, pLN and mLN when comparing *Ripk3^+/+^* and *Ripk3^−/−^* mice (Figure S3F–H, Supporting Information). Taken together, these results indicate that under carcinogen‐induced conditions, RIPK3 deficiency promotes abnormal proliferation of DP thymocytes within the thymus to initiate early‐stage of thymic tumorigenesis.

### 2.4 RIPK3 Deficiency Enhances Thymic Lymphoma in *p53* ^−/−^ Mice through ERK Hyperactivation

**Figure 4:**
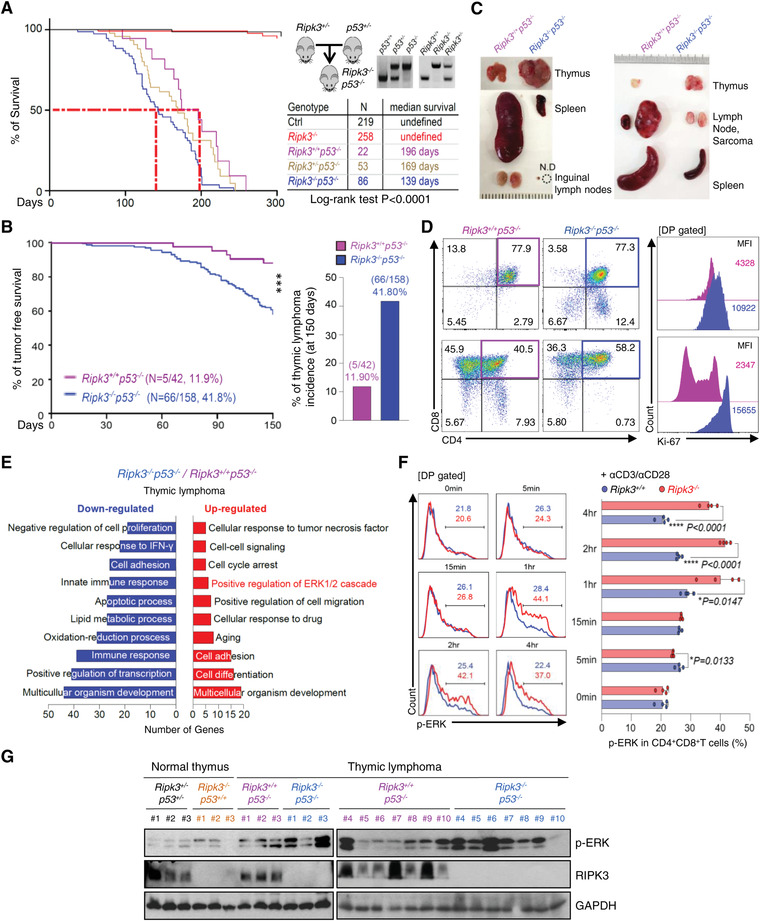
RIPK3 deficiency enhances thymic lymphoma in p53‐deficient mice via ERK hyperactivation. A) Kaplan‐Meier survival curve (Long‐rank test) of animals in different genetic backgrounds including *Ripk3^+/+^p53^−/−^* and *Ripk3^−/−^p53^−/−^* animals. Red dotted line shows median survival of *Ripk3^+/+^p53^−/−^* and *Ripk3^−/−^p53^−/‐^* mice, which are significantly different (*p* < 0.0001). B) The thymic lymphoma free survival curve and thymic lymphoma incidence of *p53^−/−^* and *Ripk3^−/−^p53^−/−^* animals until 150 days of age. The tumor‐free survival plot of *Ripk3^+/+^p53^−/−^* (*n* = 42) compared with *Ripk3^−/−^p53^−/‐^* (*n* = 158) mice that are significantly different. C) Representative images of thymus, spleen and lymph node from tumor‐bearing *Ripk3^+/+^p53^−/−^* and *Ripk3^−/−^p53^−/−^* littermates. D) The phenotype of the thymic T cell populations (left panel). The thymic T cell from both *Ripk3^+/+^p53^−/−^* and *Ripk3^−/−^p53^−/−^* thymic lymphoma showed homogenous expansion in DP T cells but *Ripk3^−/−^p53^−/−^* DP T cells showed increased proliferation index (Ki‐67) (right panel). E) RNA sequencing analysis of up‐ and down‐regulated gene in *Ripk3* ^−/−^ *p53* ^−/−^ thymic lymphoma compared to *p53* ^−/−^ thymic lymphoma. Analysis of up‐regulated gene shown the change in positive regulation of ERK signaling pathways. F) Total thymocytes from *Ripk3^+/+^* (*n* = 4) and *Ripk3^−/−^* (*n* = 4) mice were stimulated with anti‐CD3 (1 µg mL^−1^) and anti‐CD28 (2 µg mL^−1^) or without for indicated time. Phosphorylation of ERK in DP T cell measured by Flow Cytometry. G) The protein expression levels of p‐ERK in normal thymus (*Ripk3^+/−^p53^+/−^*; *n* = 3*, Ripk3^−/−^p53^+/+^*; *n* = 3) and thymic lymphoma (*Ripk3^+/+^p53^−/−^*; *n* = 10*, Ripk3^−/−^p53^−/−^*; *n* = 10) tissues were measured by Western blot analysis. Statistical analyses were performed using the two‐tailed unpaired Student *t*‐test or log rank test. *p* values below 0.05 were considered significant in the following manner: *p < 0.05, **p < 0.01, ***p < 0.001.

It is well known that *p53^−/−^* mice develop various tumors, including thymic lymphoma. ^[^ ^20^ ^]^ We also confirmed that *p53* ^−/−^ mice developed cancer in various tissues including thymus, lymph node, spleen, kidney, and liver. Notably, RIPK3 expression was significantly reduced in the thymus of mice with poor prognosis in *p53* ^−/−^ mice that developed thymic lymphoma (Figure S4A, Supporting Information). Moreover, decreased expression of RIPK3 was highly correlated with reduced survival rate in *p53* ^−/−^ mice‐bearing thymic lymphoma (Figure S4B, Supporting Information). Thus, we generated new *Ripk3^−/−^p53^−/−^* double knockout (DKO) mice to further determine whether loss of RIPK3 in *p53* ^−/−^ mice promotes thymic tumorigenesis and reduces survival. Newly crossed *Ripk3^−/−^p53^−/−^* DKO mice were viable, fertile, and normal in size without overt developmental defects. On the other hand, the overall survival of *Ripk3^+/‐^* or *Ripk3^−/‐^* mice in a *p53* ^−/−^ background was reduced compared to that of *Ripk3^+/+^p53* ^−/−^ mice with accompanying spontaneous tumorigenesis (median survival: *Ripk3^+/+^p53^−/−^* – 196 days, *Ripk3^+/−^p53^−/−^* – 169 days and *Ripk3^−/−^p53^−/−^* – 139 days) (**Figure** **4**A; Figure S4C, Supporting Information). We further analyzed a more sizable population of *Ripk3^+/+^p53^−/‐^* (N = 42) and *Ripk3^−/−^p53^−/−^* DKO (N = 158) mice and found that over a period of 150 days, *Ripk3^−/−^p53^−/−^* DKO mice showed a decreased survival rate compared to *Ripk3^+/+^p53^−/‐^* mice and a higher tumor incidence rate of thymic lymphoma (Figure 4B,C). Indeed, Ki‐67 expression was significantly increased in thymic lymphoma‐derived DP thymocytes in *Ripk3^−/−^p53^−/−^* DKO mice when compared to similar cells in *Ripk3^+/+^ p53^−/‐^* mice (Figure 4D), supporting the notion of a rapid thymic tumorigenic phenotype in *Ripk3^−/−^p53^−/−^* DKO mice. Overall, these results suggest that RIPK3 deficiency is a key factor in early tumor onset and rapid progression of thymic lymphoma in mice lacking the p53 tumor suppressor gene.

We next wanted to elucidate how RIPK3 deficiency promotes thymic lymphoma under p53 gene‐deficient conditions in DP thymocytes. We conducted RNA‐seq analyses of DP thymocytes isolated from *Ripk3^+/+^ p53^−/−^* and *Ripk3^−/−^p53^−/‐^* thymic lymphoma‐bearing mice. By gene ontology analysis, we found that ERK1/2 signaling‐related pathways were highly up‐regulated in *Ripk3^−/−^p53^−/‐^* DP thymocytes (Figure 4E). Of note, hyperactive ERK signaling is known to be a indicator in the propagation of many forms of cancer.^[^ ^21^ ^]^ Therefore, we hypothesized that RIPK3 could regulate ERK activation by a post‐transcriptional regulatory mechanism, thereby suppressing the excessive proliferation of DP thymocytes caused by abnormal ERK activation. To test this hypothesis, p‐ERK expression level analysis was performed in DP thymocytes isolated from *Ripk3^+/+^* and *Ripk3^−/−^* mice. As expected, DP thymocytes from *Ripk3* ^−/‐^ mice showed higher expression of p‐ERK in response to anti‐CD3/CD28 stimulation (Figure 4F). Indeed, p‐ERK expression levels were significantly increased in *Ripk3* ^−/−^ *p53^−/−^* thymic lymphomas compared to *Ripk3^+/+^p53^−/‐^* thymic lymphomas (Figure 4G) suggesting a critical role of RIPK3 in suppressing ERK activation.

### 2.5 LCK Interacts with RIPK3 and Phosphorylates its Tyrosine Residue

**Figure 5:**
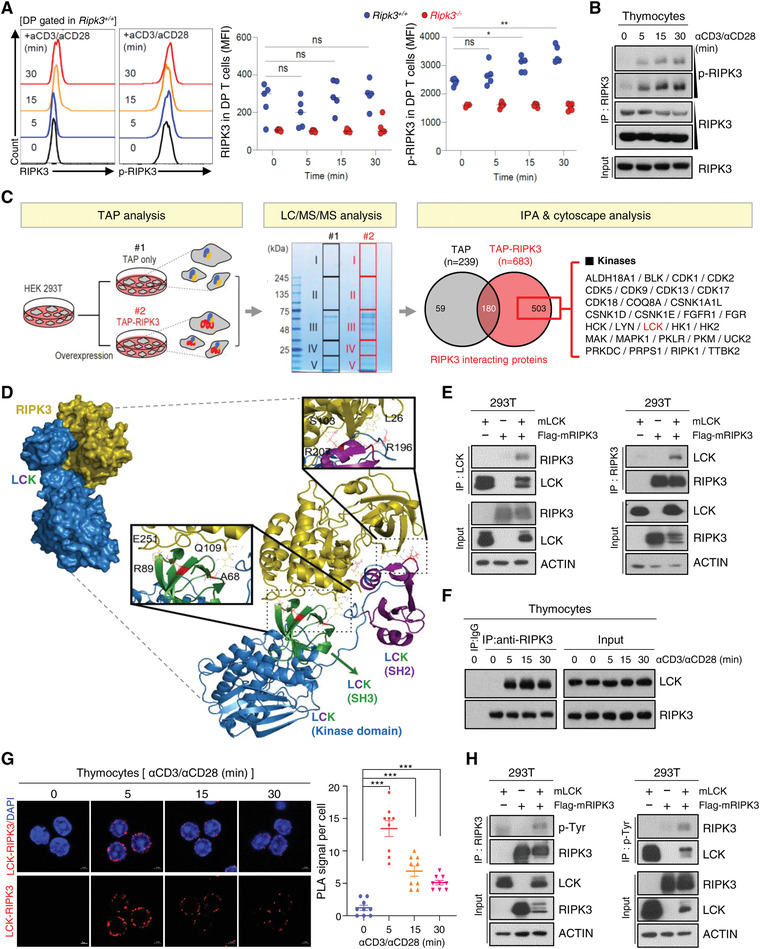
TCR‐mediated activation of LCK interacts with and RIPK3 to promote RIPK3 phosphorylation. A) Total thymocytes from *Ripk3^+/+^* (*n* = 5) and *Ripk3^−/−^* (*n* = 5) mice were cultured with anti‐CD3 (1 µg mL^−1^) and anti‐CD28 (2 µg mL^−1^) or without for indicated time. Relative fluorescence of RIPK3 and p‐RIPK3 in DP T cells. B) Phosphorylation of RIPK3 increased in anti‐CD3 (1 µg mL^−1^) and anti‐CD28 (2 µg mL^−1^) stimulation. C) Experimental workflow for TAP (Tandem Affinity purification) pull‐down assay. An aliquot of each purified sample was loaded to SDS‐PAGE and stained with Coomassie brilliant blue. Potential RIPK3‐binding proteins were identified by LC‐MS/MS. Venn diagram represents the overlap of proteins and unique proteins identified by LC/MS/MS among TAP‐purified samples as indicated. Total 503 proteins were identified as specific RIPK3 binding proteins. There were several kinases and phosphatases. D) Computational docking model for RIPK3 (olive) and LCK (blue) predicted using ClusPro (see Materials and Methods). E) Western blot analysis after immunoprecipitation of mouse RIPK3 and mouse LCK in HEK293T cells. HEK293T cells were transfected with LCK and/or Flag‐RIPK3 expression constructs. Cells were harvested at 24 h after transfection. The endogenous RIPK3 interacted with LCK in response to anti‐CD3/CD28 stimulation. Interaction of RIPK3 and LCK was observed by F) Western blot analysis, and confirmed with G) Duolink proximity ligation assay. H) Tyrosine phosphorylation of RIPK3 were detected in LCK and Flag‐RIPK3 expressing HEK293T cells. Statistical analyses were performed using the two‐tailed unpaired Student *t*‐test. *p* values below 0.05 were considered significant in the following manner: *p < 0.05, **p < 0.01, ***p < 0.001.

Next, we wished to determine how RIPK3 is involved in the regulation of DP thymocytes proliferation. Since TCR signaling is a critical determinant in DP thymocytes development and proliferation,^[^ ^22^ ^]^ we hypothesized that TCR‐mediated downstream signaling pathways may be involved in modulating RIPK3 activity, which subsequently regulates DP thymocytes homeostatic proliferation. Indeed, RIPK3 phosphorylation, but not RIPK3 protein expression, was elevated in DP thymocytes following TCR stimulation (**Figure** **5**A,B). This suggests that some TCR downstream signaling molecules might control phosphorylation‐mediated activation of RIPK3. To identify target proteins that promote phosphorylation of RIPK3, we performed a Tandem Affinity Purification (TAP) pull‐down assay ^[^ ^23^ ^]^ (Figure S5A, Supporting Information). Briefly, TAP (TAP‐only) or TAP‐tagged RIPK3 (TAP‐RIPK3) were overexpressed in HEK 293 T cells and after 24 h, each cell lysate was used for isolation of TAP‐only or TAP‐RIPK3 along with associated proteins and subsequent mass spectrometry analysis (Figure 5C left and middle panel). Excluding proteins found in TAP‐only lysates, 503 proteins were identified as potential RIPK3‐specific binding proteins. Taking advantage of network analyses: Cytoscape and Ingenuity Pathway Analysis (IPA), proteins were clustered in terms of each molecular function. Among the RIPK3‐interacting kinases, our particular interest was lymphocyte‐specific protein tyrosine kinase, or LCK, which plays important role in the development and activation of T cells by TCR‐linked signal transduction pathways (Figure 5C right panel). To evaluate the possible docking conformation of RIPK3/LCK complex, we performed a protein–protein docking simulation by using ClusPro 2.0. The scores by ClusPro for the docking model between RIP3 and LCK were low, suggesting a favorable binding mode. Briefly, four residues (Leu26/Ser103 and Gln109/Glu251) in RIPK3 formed interactions with four amino acids (Arg196/Arg 207 and Ala68/Arg 89) in SH2 and SH3 domains from LCK, supporting the structural determinants mediating protein interaction (Figure 5D; Figure S5B, Supporting Information). Co‐immunoprecipitation (Co‐IP) analyses performed by overexpressing both mouse or human LCK and RIPK3 in HEK 293T cells confirmed that these two proteins closely interact with each other (Figure 5E; Figure S5C, Supporting Information). Consistent with these findings, the interaction of LCK and RIPK3 proteins occurred markedly upon TCR activation in mouse primary thymocytes (Figure 5F). Of note, the interaction signals of these two kinases were detected in the inner leaflet of the plasma membrane of thymocytes during TCR activation, and correlated with the predominant localization of active LCK (Figure 5G).

We further interrogated the involvement of LCK in the phosphorylation of RIPK3. Although RIPK3 activity is known to be mostly regulated by phosphorylation at the sites of serine/threonine residues,^[^ ^1a^ ^]^ tyrosine 185 (Y185) phosphorylation also appears to be important in regulating the kinase activity of RIPK3.^[^ ^24^ ^]^ Therefore, we hypothesized that it might be possible for several tyrosine residues, including Y185, to be phosphorylated by active LCK in DP thymocytes. Given the lack of antibodies that distinguish each site of phosphorylated tyrosine residue of RIPK3, we instead identified a significant increase in total tyrosine phosphorylation of RIPK3 while co‐expressing LCK in HEK 293T cells (Figure 5H; Figure S5D, Supporting Information). Taken together, these results indicate that TCR‐mediated LCK activation allows active LCK to interact with RIPK3 and subsequently contribute to its phosphorylation.

### 2.6 RIPK3 Negatively Regulates ERK Phosphorylation through Potentiation of PP2A Activity

**Figure 6:**
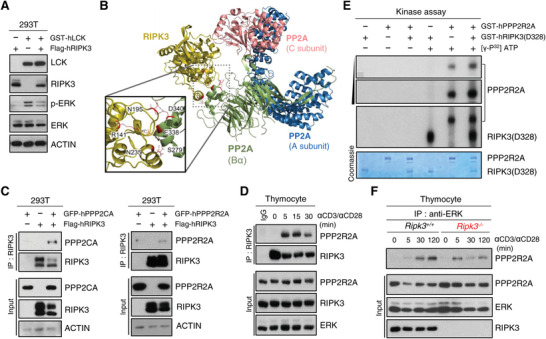
RIPK3 suppresses ERK phosphorylation by activating PP2A function. A) Western blot analysis of ERK phosphorylation after transiently expression of human RIPK3 and human LCK in HEK293T cells. HEK293T cells were transfected with GST‐LCK and/or Flag‐RIPK3 expression constructs. B) Computational docking model for RIPK3 (olive) and PP2A predicted using ClusPro. The PP2A comprises three subunits (A subunit; blue, B55*α* subunit; green, and C subunit: magenta). C) Western blot analysis after immunoprecipitation of human RIPK3 and human PP2A subunits in HEK293T cells. HEK293T cells were transfected with GFP‐PPP2CA or GFP‐PPP2R2A and/or Flag‐RIPK3 expression constructs. Cells were harvested at 24 h after transfection. D) The endogenous RIPK3 interacted with PPP2R2A in response to anti‐CD3/CD28 stimulation. E) In vitro kinase activity of RIPK3 toward PPP2R2A with ^32^P‐labeled ATP. To avoid false positive artifacts in the in vitro kinase assay, we included conditions with or without ATP. GST‐RIPK3 (amino acid 2–328) and GST‐PPP2R2A recombinant proteins were purified from sf‐9 cells. F) Total thymocytes from *Ripk3^+/+^* and *Ripk3^−/−^* mice were stimulated with anti‐CD3 (1 µg mL^−1^) and anti‐CD28 (2 µg mL^−1^) for indicated time. Cell lysates were immunoprecipitated with anti‐ERK antibody. Immunocomplexes and cell lysates were analyzed by immunoblotting.

ERK is known to phosphorylated downstream of LCK‐mediated signaling, however, the p‐ERK level was significantly reduced in the presence of both LCK and RIPK3 (**Figure** **6**A), suggesting a critical role for RIPK3 in suppressing LCK‐mediated ERK activation. Thus, we wished to assess how activated RIPK3 represses ERK activation. From our TAP‐MASS analysis, we identified PPP2R2A (B55*α* regulatory subunit of PP2A), a known phosphatase of ERK,^[^ ^25^ ^]^ as a RIPK3 interacting protein (Figure S6A, Supporting Information). As a phosphatase, PP2A is ubiquitously expressed in eukaryotic cells and exists as a heterotrimeric enzyme composed of a catalytic C subunit, a scaffolding A subunit and multiple regulatory B subunits that are thought to regulate enzyme activity.^[^ ^26^ ^]^ It has been reported that PKA activates PP2A via phosphorylation of the B56 subunit, which is one of the regulatory subunits of PP2A.^[^ ^27^ ^]^ Based on these previous notions, we tested the functional properties of RIPK3 as a potential kinase regulator of PP2A. First, we performed a protein–protein docking simulation and found that three residues (Arg141, Asn195, and Asn235) of RIPK3 protein interacted with three amino acids (Ser279, Glu338, and Asp340) of the PP2A B55*α* subunit (PPP2R2A) (Figure 6B; Figure S6B, Supporting Information). Indeed, immunoprecipitation studies demonstrated that RIPK3 interacts with the regulatory (PPP2R2A) subunit as well as the catalytic (PPP2CA) subunit of PP2A (Figure 6C). Consistent with these findings, the PPP2R2A and RIPK3 protein interaction occurred markedly upon TCR activation in mouse primary thymocytes (Figure 6D), suggesting the possibility that RIPK3 could regulate phosphatase activity of PP2A via phosphorylation of its regulatory subunit.

To further test this hypothesis, we performed in vitro kinase assay using [*γ*‐^32^P] ATP and visualized by autoradiography. Importantly, the ^32^P labeled‐PPP2R2A band appeared stronger in the presence of RIPK3 (Figure 6E; Figure S6C, Supporting Information), however, the phosphorylation status of RIPK3, especially at serine 227, was not affected by PP2A overexpression (Figure S6D, Supporting Information), indicating that PPP2R2A was phosphorylated as a specific substrate of RIPK3. Moreover, in response to anti‐CD3/CD28 stimulation, the interaction between ERK and PPP2R2A was significantly reduced in the absence of RIPK3 in immunoprecipitation studies, suggesting that RIPK3 is an important mediator in regulating the interaction of PPP2R2A with its substrate, ERK (Figure 6F). Overall, these results indicate that RIPK3 serves as an important regulator in modulating ERK activation by promoting the phosphatase activity of PP2A as well as its interaction with ERK.

### 2.7 Pharmacological Modulation of PP2A Activity Regulates DP Thymocyte Proliferation in a Carcinogen‐Induced Mouse Model

**Figure 7:**
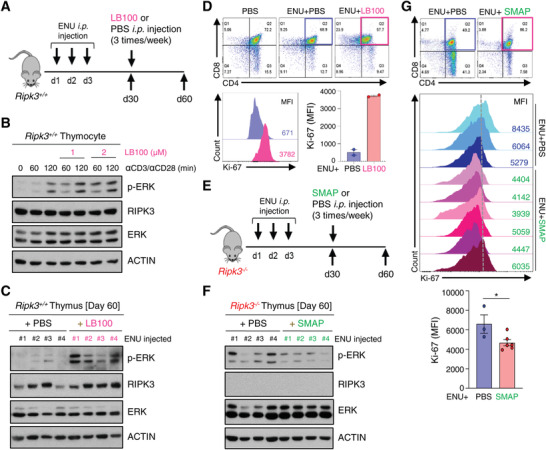
Pharmacological modulation of PP2A activity regulates DP thymocyte hyperproliferation‐associated tumorigenesis. A) Intraperitoneal injection of ENU at around postnatal day 13 ∼15 (three‐time) was performed to *Ripk3^+/+^* mice. After 30 days of injection, mice were injected with LB‐100 or PBS intraperitoneally at 2 mg kg^−1^ on alternate days for 30 days. (*n* = 4 for each group). B) Phosphorylation of ERK increased in response to anti‐CD3 (1 µg mL^−1^) and anti‐CD28 (2 µg mL^−1^) stimulation, which was further increased by LB‐100 treatment. C) Activation of ERK increased in LB‐100 injected *Ripk3^+/+^* thymus. D) Relative fluorescence of Ki‐67 in thymic T cells analyzed by Flow Cytometry. Ki‐67 expression is enhanced in LB‐100 injected *Ripk3* ^+/+^ thymocytes. E) Schematic diagram of an ENU injection experiment. After 30 days of ENU injection, *Ripk3^−/−^* mice were injected with SMAP or PBS intraperitoneally at 2 mg kg^−1^ on alternate days for 30 days. (*n* = 4 – 6 for each group). F) Activation of ERK reduced in SMAP injected *Ripk3^−/−^* thymus. G) Ki‐67 expression is decreased in SMAP injected *Ripk3^−/−^* thymocytes. Relative fluorescence of Ki‐67 expression analyzed by Flow Cytometry. Statistical analyses were performed using the two‐tailed unpaired Student *t*‐test. P values below 0.05 were considered significant in the following manner: *p < 0.05, **p < 0.01, ***p < 0.001.

To determine the functional importance of PP2A in regulating ERK activation of DP thymocytes in the context of thymic lymphoma, we used a selective PP2A inhibitor, LB‐100 that is suitable for in vivo via intraperitoneal injection in an ENU‐induced tumor model (**Figure** **7**A). First, we confirmed that LB‐100 inhibited ERK activation in response to TCR activation in *Ripk3* ^+/+^ DP thymocytes (Figure 7B; Figure S7A, Supporting Information). Although injection of LB‐100 did not significantly affect the thymus phenotype over a short period of time after ENU‐injection in *Ripk3* ^+/+^ mice (Figure S7B, Supporting Information), the activation of ERK was further enhanced in *Ripk3* ^+/+^ DP thymocytes injected with LB‐100 in comparison with those injected with PBS (Figure 7C). Furthermore, Ki‐67 expression is enhanced in LB‐100 injected *Ripk3* ^+/+^ DP thymocytes (Figure 7D), suggesting that loss of PP2A activity promotes abnormal proliferation of DP thymocytes. As expected, administration of LB‐100 did not alter the expression pattern of PP2A, suggesting that it only affects its phosphatase activity (Figure S7C). Next, we wished to investigate whether rescue of PP2A activity could suppress the ERK activation in RIPK3‐deficient DP thymocytes in the context of carcinogen‐induced tumorigenesis (Figure 7E). We used a PP2A activator, SMAP which is known to bind the scaffold subunit alpha of PP2A and promote its phosphatase activity by causing conformational change.^[^ ^28^ ^]^ As expected, ERK activation was decreased in RIPK3‐deficient DP thymocytes injected with SMAP without any alteration of the expression pattern of PP2A (Figure 7F; Figure S7D, Supporting Information), which in turn significantly decreased the level of Ki‐67 (Figure 7G). Administration of SMAP rescued thymus size and pLN in the context of carcinogen‐induced tumorigenesis (Figure S7E, Supporting Information). Collectively, these results suggest the possibility that modulation of PP2A activity may regulate RIPK3‐mediated initiation and the development of thymic lymphoma.

## 3 Discussion

While most previous studies of the RIPK3 protein have been mainly focused on its pro‐necroptotic functions, it has been less than clear as to how RIPK3 may function in tumorigenesis. We demonstrated here that RIPK3 deficiency accelerated tumor progression and decreased survival in a carcinogen‐induced tumorigenesis model and p53 gene‐deficient background. This is consistent with a recent study showing that when *Ripk3^−/−^* mice are crossed with *Apc^Min/+^* mice carrying APC gene mutations (which induce spontaneous development of intestinal tumors), the progeny exhibits increased tumor formation.^[^ ^9b^ ^]^ Through long‐term follow‐up, we found that various types of spontaneous cancers were developed with high frequency in *Ripk3^−/−^* mice (20%) than *Ripk3^+/+^* mice (5.9%), particularly liver, lung, and thymus. Previous animal models with germline deletion of the *Ripk3* gene did not report spontaneous tumor formation in these studies.^[^ ^18^ ^]^ There are two main reasons why our observations somewhat differ from these previous studies. First, most previous studies observed phenotypes at early time points. For instance, in the study by Newton et al.,^[^ ^18a^ ^]^ which first described the RIPK3 phenotype the timeframe in which the mouse phenotype was observed and analyzed in order to demonstrate the function of RIPK3 in terms of necroptosis and inflammation was relatively short compared to our present study. Second, in all previous RIPK3 mouse studies, the mouse models were exclusively maintained in the C57BL/6 (B6) genetic background. However, the animals we analyzed were maintained in a mixed genetic background of B6 and 129/Sv [B6/129]. As scientific initiative of the current study was to look at the possible involvement of RIPK3 in tumorigenesis, we believe switching from a genetic background of B6 to B6/129 was appropriate to answer our questions with regard to RIPK3 effects on tumorigenesis. As described in our initial version of the manuscript, we found quite a high incidence of thymic lymphoma formation in *Ripk3^−/−^p53^−/−^* mice. Then we reasoned that a *Rikp3* null condition could provide the favorable statue to be a transformation of the thymus. That is why we analyzed the *Rikp3^−/−^* thymus whose genetic background was fully shifted to a B6/129 background. Our data, therefore suggest that for observing the function of RIPK3 on tumor suppression, especially thymic lymphoma, the effects on tumor development are more easily observed in a tumor‐prone genetic background, with a long‐term in vivo model analysis method.

More importantly, we noted for the first time that the canonical RIPK1‐RIPK3‐MLKL necroptosis pathway is not involved in thymic lymphoma development. The thymus is the primary site of T cell development but both T and B cell lymphoma can arise in the thymus.^[^ ^29^ ^]^ The most common thymic lymphoid neoplasms are Hodgkin's lymphoma, large‐cell lymphoma, and lymphoblastic lymphoma.^[^ ^30^ ^]^ Extensive loss/reduction of RIPK3 expression in many cancers suggests its loss provides some kind of selective advantage to tumor cells and a defect of canonical necroptosis could be a central reason for tumor progression/tumor cell proliferation.^[^ ^9a^ ^]^ However, we found that MLKL was hardly expressed in DP T lymphocytes from the thymus and in thymic lymphoma cells. This suggests that RIPK3 functions in regulating DP thymocytes population independently of the canonical RIPK3‐MLKL necroptosis pathway. In the future, it would be interesting to elucidate the mechanism by which the expression of MLKL is suppressed in DP thymocytes. However, it is tempting to speculate, given our findings that this suppression may protect thymocytes from TCR‐mediated necroptosis while RIPK3 is regulating the homeostatic population of thymocytes via another pathway.

The mechanism for engagement of RIPK3 in maintaining of thymic T cell homeostasis was demonstrated by finding of LCK as a binding partner of RIPK3 through TAP‐MASS and protein–protein docking simulation experiments. This finding was further supported by data showing that LCK and RIPK3 interaction occurred markedly upon TCR activation in thymocytes and LCK promotes RIPK3 kinase activity through phosphorylation of RIPK3 in DP thymocytes. However, specific tyrosine residues of RIPK3 involved in kinase activity by LCK were not considered. In line with this, we further demonstrated that LCK‐mediated RIPK3 activation inhibits thymic lymphoma development through the attenuation of ERK activation. TCR‐linked ERK activation has been implicated in the positive and negative selection of T lymphocytes in the thymus as well as in the development of thymic lymphoma initiation and progression.^[^ ^31^ ^]^ ERK phosphorylation was further increased in DP thymocytes of Ripk3−/− mice bearing thymic lymphoma, indicating that RIPK3 deficiency results in loss of regulation for ERK activation. The phosphorylation status of ERK can be modulated by the phosphatase PP2A, and PP2A was identified as a downstream target of RIPK3. PP2A activity is known to be regulated by its phosphorylation status on its regulatory subunit and RIPK3 was able to phosphorylate the PP2A regulatory subunit, however, we did not identify the specific amino acid that was phosphorylated. Our findings now add a post‐transcriptional layer of control on ERK signaling mediated by the LCK‐RIPK3‐PP2A axis in DP thymocytes, thereby maintaining appropriate ERK activation in this context.

There are various pharmacological drugs that target PP2A and are applied to embryonic stem cell pluripotency, cell viability, and cancer therapy,^[^ ^32^ ^]^ raising the possibility that these compounds might be repurposed to ameliorate thymic lymphoma progression dependent on the PP2A‐ERK axis. Based on the results of a PP2A agonist inhibiting ERK activation and the proliferation of DP thymocytes in a carcinogen‐induced tumor model, a targeting this axis may also be useful to reprogram PP2A‐ERK axis and enhance the efficacy of limiting tumorigenic DP thymocyte proliferation in thymic lymphoma. Necroptosis‐independent functions of RIPK3 may be driving its loss in cancer by providing accretion of tumor progression in concert with increased oncogenic signaling such as ERK. Our study thus provides the evidence demonstrating the tumor suppressive function of RIPK3 and suggests that the measurement of RIPK3 levels may be clinically relevant for tumor prognosis and therapy.

## 4 Conclusion

This study uncovers that RIPK3‐PP2A‐ERK axis is the key regulatory pathway in maintaining DP thymocytes homeostasis. Ablation of this axis sensitizes thymic tumorigenesis in mice exposed to carcinogens or mice depleted of tumor suppressor genes. Thus, revisiting the unidentified role of RIPK3 as a tumor suppressor could lead to new therapeutic modalities targeting cancer patients.

## 5 Experimental Section

### Mouse Strains

*Ripk3^−/‐^* mice carrying a germline deletion of *Ripk3* were kindly provided by Dr. V. M. Dixit (Genentech, USA). *p53^−/−^* mice were originally obtained from Jackson Laboratory. In brief, *Ripk3^+/‐^* mice were bred each other to generate *Ripk3^+/+^, Ripk3^+/‐^* and *Ripk3^−/−^* mice, respectively; *Ripk3^+/+^ p53^+/+^, Ripk3^+/−^p53^+/‐,^ Ripk3^+/−^p53^+/‐^* and *Ripk3^−/−^p53^−/−^* mice were generated in similar fashion by breeding *Ripk3^+/−^p53^−/−^* mice each other. All mice were maintained in the Laboratory Animal Research Center (LARC) of Ajou University Medical Center in Specific Pathogen Free Condition. Mixtures of male and female mice were used, matched between groups. Mice were sacrificed between the age of 5–7 weeks. In the case of survival curve and tumor study, mice were kept till over 25 to less than 60 weeks. Mice were observed for any sign of sickness/discomfort and, if it was necessary, palpation was applied to examine the tumor mass inside the body. The end point of each mice with tumors was determined humanely. All experimental procedures were approved by the Laboratory Animal Research Center (LARC) of Ajou University Medical Center Institutional Animal Care and Use Committee (IACUC), who approved all animal procedures (2014‐0056, 2020‐0026).

### Cell Culture

For mice samples, single‐cell suspensions from thymus, spleen, peripheral lymph nodes, mesenteric lymph nodes were maintained in complete T cell medium; RPMI medium supplemented with 10% fetal bovine serum (FBS), 1% penicillin‐streptomycin, 2 mm L‐glutamine, 1 mm Sodium Pyruvate, 10 mm HEPES, 1X MEM Non‐Essential Amino Acids and 0.1% 2‐Mercaptoethanol. *Ripk3^−/−^* mouse embryonic fibroblast (MEF) cells and their culture conditions had been previously described. ^[^ ^33^ ^]^ Primary thymic cells were L5180, L1210, and RAW264.7 cells were maintained in RPMI medium supplemented with 10% FBS and 1% penicillin‐streptomycin. EL‐4 and 293T cells were cultured in DMEM medium supplemented with 10% FBS and 1% penicillin‐streptomycin.

### Flow Cytometric Analysis

For mice samples, single‐cell suspensions, which had been cultured in the absence and/or presence of anti‐CD3 (1 µg mL^−1^; 100 302, 145‐2C11, BioLegend) and anti‐CD28 (2 µg mL^−1^; 102 102, 37.51, BioLegend), from thymus, spleen, peripheral lymph nodes, mesenteric lymph nodes were prepared and stained with Fixable Viability Dye (Zombie NIR Fixable Viability Kit, 423 106, BioLegend) to label dead cells and stained with fluorochrome‐conjugated antibodies. For surface staining, cells were washed with PBS and stained with the following antibodies (From BioLegend): anti‐CD4 (GK1.5), anti‐CD8 (53‐6.7), anti‐CD11b (M1/70), anti‐CD11c (N418), anti‐CD86 (GL‐1), anti‐CD206 (C068C2), anti‐CD25 (3C7), anti‐CD44 (IM7), anti‐CD62L (MEL‐14). For intracellular staining, cells were fixed with eBioscience/Invitrogen Foxp3 Fix/Perm Buffer washed with eBioscience/Invitrogen Perm Buffer and stained with the following antibodies: anti‐p‐ERK1/2 (p44/42) (9102, Cell Signaling Technology), anti‐Ki‐67 (15 580, Abcam), anti‐RIPK3 (ADI‐905‐242‐100, Enzo), anti‐p‐RIPK3 (91 702, Cell Signaling Technology) and anti‐MLKL (ab184718, Abcam). For detection of cellular necroptosis, single‐cell suspensions from thymus were cultured under necroptotic stimulus conditions (TNF‐*α* (30 ng mL^−1^; R&D Systems), SMAC mimetic (200 nm; Adooq Bioscience), zVAD (20 µm, Enzo)). FITC Annexin V Apoptosis Detection Kit I (BD Bioscience) was used, following the manufacturer's instructions. Cell acquisition was performed on FACS Canto II (BD Bioscience) and data were analyzed using FlowJo software suite (Treestar).

### Immunohistochemistry

For histology, thymus and spleen from mice were excised and fixed 12 h in 10% formalin solution at 4 °C. Tissues were embedded in paraffin, cut into 5 µm sections (LEICA, HistoCore MULTICUT), deparaffinized and dehydrated via sequential addition of xylene, 100% ethanol, 95% ethanol, 85% ethanol, and 70% ethanol. The sections were then washed in distilled water and stained with Hematoxylin (S330930‐2, Agilent) and Eosin (32 002, MUTO). Mounting solution (9 999 122, Thermo Fisher Scientific) dropped on the samples and dry at 25 °C for 12 h. Localization of immune‐positive signals was visualized using DAB (diaminobenzidine, TA‐125‐HDX, Thermo Fisher Scientific) staining method. The primary antibodies used for this study were RIPK3 (ADI‐905‐242‐100, Enzo), Ki‐67 (ab15580, Abcam). All processed slides were examined and imaged using a B600TiFL (Optika) equipped with a DFC310 FX digital camera (Leica). Microscopic images were processed using Photoshop (v CS6.5, Adobe).

### Proximal Ligation Assay

Thymocytes were stimulated with anti‐CD3 (1 µg mL^−1^) and anti‐CD28 (2 µg mL^−1^) for indicated time, then cells were seeded on coverslips. Coverslips were pretreated with poly‐L‐lysine solution (P8920, Sigma) diluted in PBS (1:20) at 37 °C for 2 h prior to cell seeding. Cells were fixed in 4% paraformaldehyde for 20 min followed by permeabilization with 0.25% Triton‐X 100 for 15 min. Proximal ligation assay (PLA) Duolink kit (DUO92102, Sigma) instructions were followed, after incubation in the Duolink blocking solution for 1 h at 37 °C, the cells were incubated 12 h at 4 °C with the following primary antibodies: anti‐LCK (sc‐433, Santa Cruz Biotechnology) and anti‐RIPK3 (NBP1‐77299, Novus). Then, they were incubated with the Duolink PLA probe for 1 h at 37 °C, wash the slides and incubate with the ligase in a pre‐heated humidity chamber for 30 min at 37 °C. After incubation, add polymerase with the slides for 100 min at 37 °C. A mounting medium containing DAPI was used for counterstaining.

### Western Blot

Cells from each group were lysed using M2 buffer ^[^ ^34^ ^]^ and mice tissue were lysed using a lysis buffer composed of 50 mm Tris‐HCl (pH 7.5), 150 mm NaCl, 50 mm NaF, 1% Tween 20, 0.2% NP‐40 and protease inhibitors. Equal amounts of cell and tissue extracts were separated by SDS‐PAGE. Proteins were detected using the following antibodies. Primary antibodies: anti‐RIPK1 (610 459, BD Bioscience), anti‐Mouse‐RIPK3 (ADI‐905‐242‐100, Enzo), anti‐Human‐RIPK3 (13 526, Cell Signaling Technology), anti‐Mouse‐p‐RIPK3 (91 702, Cell Signaling Technology), anti‐Human‐p‐RIPK3 (ab209384, Abcam), anti‐MLKL (ab184718, Abcam), anti‐GAPDH (25 778, Santa Cruz Biotechnology), anti‐ACTIN (sc4778, Santa Cruz Biotechnology), anti‐VINCULIN (V9131, Sigma–Aldrich), anti‐ERK (9102, Cell Signaling Technology), anti‐p‐ERK (9101, Cell Signaling Technology), anti‐LCK (sc‐433, Santa Cruz Biotechnology), anti‐p‐LCK (Y393) (ab208787, Abcam), anti‐p‐Tyr (9411, Cell Signaling Technology), anti‐p‐Tyr (8954, Cell Signaling Technology), anti‐PPP2R2A (5689, Cell Signaling Technology), anti‐PPP2CA (610 555, BD Bioscience), anti‐GST (27‐4577, GE Healthcare) and anti‐FLAG (F1804, Sigma–Aldrich). Secondary antibodies: Peroxidase‐conjugated AffiniPure Goat Anti‐Rabbit IgG (111‐035‐003, Jacson ImmunoResearch), Peroxidase‐conjugated AffiniPure Sheep Anti‐Mouse IgG (515‐035‐003, Jacson ImmunoResearch). Images were detected using enhanced chemiluminescence (Pierce ECL Western blotting substrate, 32 106, Thermo) and film (AGFA, EA8EC).

### Immunoprecipitation

Lysates were mixed and precipitated with antibody and protein A‐sepharose or protein G‐agarose beads 12 h at 4 °C. Bound proteins were removed by boiling in SDS and separated by SDS‐PAGE and immunoblotting visualized by enhanced chemiluminescence (Pierce ECL Western blotting substrate, 32 106, Thermo).

### In Vivo ENU‐Induced Tumor Model

In carcinogen‐induced mouse model, *N*‐ethyl‐*N*‐nitrosourea (ENU) (759‐73‐9, Sigma–Aldrich) or PBS was administrated intraperitoneally (0.5 µmol per g of body weight) to *Ripk3^+/+^* and *Ripk3^−/‐^* mice at postnatal day 14 (P14). After 30 days of injection, mice were injected with LB‐100 (S7537, Selleck) or SMAP (S8774, Selleck) intraperitoneally at 2 mg kg^−1^ on alternate days for 30 days.

### RNA‐seq Analysis

Thymus were excised from *Ripk3^+/+^p53^−/−^* (Thymic lymphoma), *Ripk3^−/−^p53^−/−^* (Thymic lymphoma) mice. Three replicates of each group were prepared. RNA was isolated from each thymus using RNeasy Mini Kit (74 106, Qiagen) according to manufacturer's instruction. RNA‐Seq samples were analyzed using TopHat–Cufflinks pipeline. Reads were mapped to the mm10 genome with TopHat v2.1.1. Transcripts were assembled using Cufflinks v2.2.1. Final transcriptome assembly was performed with cuffmerge v1.0.0, and differential expression was identified with cuffdiff v2.2.1. Gene ontology analysis for gene sets was performed using Database for Annotation, Visualization and Integrated Discovery (DAVID, v6.8). The GEO accession number for the raw and processed RNA‐Seq data reported in this article is GSE213606.

### Tandem Affinity Purification and Mass Spectrometry (TAP‐MS) Analysis

The InterPlay Mammalian TAP System protocol (Q‐240107, Agilent Technologies) was followed. In brief, HEK293T cells were cultured in high glucose‐DMEM medium supplemented with 10% FBS and 1% penicillin‐streptomycin. For isolation of RIPK3‐binding proteins, HEK293T cells were transfected with the pNTAP empty vector or pNTAP‐RIPK3 and pNTAP‐RIPK3 K50A vector using PEI (Polyetherimide). After 24 h post‐transfection, cells were lysed by lysis buffer supplemented with protease inhibitor cocktail and 1 mm phenylmethylsulfonyl fluoride (PMSF), and centrifuged at a speed of 16 000 g for 10 min at 4 °C. The supernatants were collected, and the cell lysates were mixed with 50 µL (50% slurry) of streptavidin resin and incubated at 4 °C for 2 h on a rotator. After centrifugation at 1500 g for 5 min, the supernatants were discarded, and the collected resins were washed in streptavidin binding buffer three times. TAP‐tagged RIPK3 and its binding proteins were eluted with streptavidin‐elution buffer at 4 °C for 30 min on a rotator. Eluted proteins were loaded by SDS‐PAGE and stained using a gel code staining kit (Thermo Fisher Scientific). The molecular identities of eluted proteins were analyzed by mass spectrometry.

### Protein Structure and in Silico Binding Analysis

Human protein phosphatase 2A (PP2A) protein structure (PDB ID: 3DW8) was retrieved from Protein Data Bank (http://www.rcsb.org). Homology‐based structural modeling of RIPK3 (accession ID: NP_006862.2) and LCK (accession ID: NP_005347.3) was performed using the SWISS‐MODEL web server (http://swissmodel.expasy.org).^[^ ^35^ ^]^ The templates, mouse RIPK3 (PDB ID: 4M69) and human tyrosine kinase (PDB ID: 1Y57), were selected for RIPK3 and LCK (the sequence similarities are 69.7% and 60.3%). The QMEAN4 Z‐scores given by SWISS‐MODEL were −2.12 and −0.55. Computational docking simulations were conducted with ClusPro 2.0 using the hydrophobic‐favored scoring scheme.^[^ ^36^ ^]^ The scores by ClusPro 2.0 for the docking model were −983.9 and −796.5.

### In Vitro Kinase Assay

For purifying recombinant human GST‐RIPK3 kinase domain (amino acids [aa] 2–328) and GST‐PPP2R2A protein derived from insect cells. Prepare for these proteins, DH10Bac *E. coli* were transformed with pDEST20 to generate recombinant bacmid DNA. Sf9 cells were transfected with purified bacmid DNA. For protein expression, Sf9 cells were infected with baculovirus and after 5 days later, cells were collected and lysed with NETN buffer (25 mm Tris‐HCl (pH 8.0), 150 mm NaCl, 1 mm DTT, 1% NP‐40, 0.1% Triton X‐100 and protease inhibitor). The soluble fraction was separated by centrifugation and incubated with glutathione Sepharose 4B beads at 4 °C for 3 h. The protein‐bound beads were washed with NETN buffer and eluted with elution buffer (50 mm HEPES (pH 7.5), 100 mm NaCl, 10% glycerol, 40 mm L‐glutathione reduced). Purified baculovirus‐expressed recombinant human RIPK3 (amino acids [aa] 2–328) and PPP2R2A were incubated with kinase buffer (100 mm NaCl, 2 mm MgCl_2_, 2 mm MnCl_2_, 1 mm DTT, 20 mm HEPES, 10 µg mL^−1^ Leupeptin and 1 X phosphostop) and [*γ*‐^32^P] ATP for 30 min at 30 °C. After incubation, samples were boiled and separated by SDS‐PAGE. Kinase activity was determined by radiography.

### Statistical Analysis

For statistical analyses, all experiments were performed more than three times. Statistical analyses were performed using the two‐tailed unpaired Student *t*‐test or log rank test (Kaplan–Meier curves for overall tumor free‐ or bearing‐survival) through GraphPad Prism 8 program. *p* values below 0.05 were considered significant in the following manner: *p < 0.05, **p < 0.01, ***p < 0.001. Within bar graphs, bars represent means while error bars indicate SEM.

## Conflict of Interest

The authors declare no conflict of interest.

## Author Contributions

S‐M.H. and Y‐J.H. performed most of the experiments, analyzed the data, visualized, and wrote the manuscript. G‐B.K., H‐J.N., A‐Y.L., B‐J.K., and S.M.H., participated in experiments. J.R. provided technical support for RNA‐Seq experiments. D.L. analyzed Immuno‐histochemistry data and pathology of tumor tissues. S‐I.Y. performed the computational analysis of protein structures and protein‐protein interactions. M.J.M. and Y.L. provided technical and intellectual support for the analysis of in vivo data. Y‐S.K. wrote the manuscript and supervised this study and takes responsibility for the data. All authors contributed to the article and approved the final version of the manuscript.

## Supporting information

Supporting Information

## Data Availability

The data that support the findings of this study are available from the corresponding author upon reasonable request.
