# Supplementary material for: LCK‐Mediated RIPK3 Activation Controls Double‐Positive Thymocyte Proliferation and Restrains Thymic Lymphoma by Regulating the PP2A‐ERK Axis
Source: Adv Sci (Weinh). 2022 Sep 25;9(32):2204522. doi: 10.1002/advs.202204522 (PMC9661840; doi:10.1002/advs.202204522)
Supplement: Supplementary file 1 — Supporting Information [file ADVS-9-2204522-s001.pdf]

LCK-mediated RIPK3 Activation Controls Double-Positive Thymocyte Proliferation and Restrains Thymic Lymphoma by Regulating the PP2A-ERK Axis

Sung-Min Hwang[#], Yu-Jin Ha[#], Gi-Bang Koo, Hyun-Jin Noh, A-Yeon Lee, Byeong-Ju Kim, Sun Mi Hong, Michael J. Morgan, Seong-il Eyun, Dakeun Lee, Jae-Seok Roe, Youngsoo Lee, and You-Sun Kim[*]

S. Hwang, Y. Ha, G. Koo, H. Noh, A. Lee, B. Kim, S. Hong, Y. Kim
Department of Biochemistry and Molecular Biology, Ajou University School of Medicine, 164 Worldcup-ro, Yeongtong-gu, Suwon, Gyeonggi-do, 16499, Republic of Korea.

S. Hwang
Sandra and Edward Meyer Cancer Center and Department of Obstetrics and Gynecology, Weill Cornell Medicine, New York, NY, 10065, USA.

Y. Ha, G. Koo, H. Noh, A. Lee, B. Kim, Y. Kim
Department of Biomedical Sciences, Graduate School of Ajou University, 164 Worldcup-ro, Yeongtong-gu, Suwon, Gyeonggi-do, 16499, Republic of Korea.

M. J. Morgan
Department of Natural Sciences, Northeastern State University, Tahlequah, OK, 74464, USA.

S. Eyun
Department of Life Science, Chung-Ang University, Seoul, 06973, Republic of Korea

D. Lee
Department of Pathology, Ajou University School of Medicine, 164 Worldcup-ro, Yeongtong-gu, Suwon, Gyeonggi-do, 16499, Republic of Korea.

J. Roe
Department of Biochemistry, College of Life Science and Biotechnology, Yonsei University, Seoul, 03722, Republic of Korea

Y. Lee
Institute of Medical Science, Ajou University School of Medicine, 164 Worldcup-ro, Yeongtong-gu, Suwon, Gyeonggi-do, 16499, Republic of Korea.

Y. Kim
Lead Contact

# These authors contributed equally to this work.

*To whom correspondence should be addressed.

You-Sun Kim, Ph.D.

Department of Biochemistry and Molecular Biology, Ajou University School of Medicine, 164 Worldcup-ro, Yeongtong-gu, Suwon, Gyeonggi-do, 16499, Republic of Korea
Tel: 82-31-219-4509, Fax: 82-31-219-4530
E-mail: yousunkim@ajou.ac.kr

KEYWORDS

RIPK3, Thymic lymphoma, Double-positive (DP) thymocytes, LCK, PP2A, ERK

Supporting Information Figure Legends

Supporting information 1. Analysis of thymus- and spleen-derived immune cells from RIPK3-deficient mice.

A) The expression level of RIPK3 protein in the thymus (left panel) and spleen (right panel). RIPK3 protein level measured by Western blot analysis.

B) Thymi from *Ripk3*$^{+/+}$ and *Ripk3*$^{-/-}$ mice are shown.

C) Populations of dendritic cells (CD11c^{+}) and macrophages (F4/80^{+}) in thymic T cells. (n=7 for each group).

D) Spleen from *Ripk3*$^{+/+}$ and *Ripk3*$^{-/-}$ mice is shown (left) and its weight is shown in the graph (right). (n=5 for each group).
E) Representative H&E images of spleen from both *Ripk3*$^{+/+}$ and *Ripk3*$^{-/-}$ mice. (R: red pulp. W: white pulp).

F) Splenic T cell populations from *Ripk3*$^{+/+}$ and *Ripk3*$^{-/-}$ mice. Average of the splenic T cell subsets and the absolute cell number by the indicated subsets are shown in each graph. (n=7 for each group).

G) Populations of naïve (CD62L^{hi}CD44^{lo}) and effector T cell (CD62L^{lo}CD44^{hi}) in the splenic T cells. (n=7 for each group).

H) Populations of dendritic cells (CD11c^{+}) and macrophages (CD11c^{-}F4/80^{lo}, CD11c^{-}F4/80^{hi}) in splenic T cells. (n=7 for each group).

Statistical analyses were performed using the two-tailed unpaired Student *t*-test. P values below 0.05 were considered significant in the following manner.

Supporting information 2. RIPK1, RIPK3 and MLKL expression levels in various tissues and different cell types.

A) Total thymocytes from *Ripk3*$^{+/+}$ (n=6) and *Ripk3*$^{-/-}$ (n=6) mice were treated with TNF-α (50 ng/ml), z-VAD (20 μM) and Smac mimetic (200 nM) for 5 h and 24 h. Dead cells were identified by Annexin V and PI staining.

B-D) Distribution of *Ripk1* (B), *Ripk3* (C) and *Mlkl* (D) gene expression in various immune

cell from BioGPS website.

E, F) Expression levels of MLKL in the indicated cell lines. MLKL protein level was measured by Western blot analysis (E) and Flow Cytometry (F).

Supporting information 3. Loss of RIPK3 does not impact the population and proliferation of T cells from spleen and peripheral lymph nodes in carcinogen-induced mouse model.

A) The ratio of thymus weight from control mice and ENU-injected mice were shown in the graph.

B) Spleen (left), peripheral lymph node (pLN; middle) and mesenteric lymph node (mLN; right) of each genotype is shown.

C-H) T cell populations of spleen (C), pLN (E) and mLN (G) from the indicated genotype, displayed as 2D plots of relative fluorescence of the indicated marker. Average of the T cell subsets are shown in each graph. Relative fluorescence of Ki-67 in $CD4^{+}$ and $CD8^{+}$ T cells. (C, D; Spleen, E, F; pLN, G, H; mLN). *Ripk3*$^{+/+}$ and *Ripk3*$^{-/-}$ littermates were analyzed at 60 days after three-time injections of ENU (n=10 -12 for each group).

Statistical analyses were performed using the two-tailed unpaired Student *t*-test. P values below 0.05 were considered significant in the following manner.

Supporting information 4. Tumor progression and incidence rate according to RIPK3 expression in thymic lymphoma-bearing Trp53-deficient mice

A, B) Western blotting analysis of RIPK3 expression level in tissues lysates from *p53*$^{-/-}$ background thymic lymphoma.

C) Tumors found in various organs and their frequency in *Ripk3*$^{+/+}$*p53*$^{-/-}$ and *Ripk3*$^{-/-}$*p53*$^{-/-}$ animals.

Supporting information 5. Protein Interacting domains between LCK and RIPK3

A) Tandem affinity purification of the tagged RIPK3 protein and interacting proteins using

streptavidin resin followed by calmodulin resin.

B) Amino acid sequence of human LCK (upper panel) and human RIPK3 (lower panel).

C, D) Western blot analysis after immunoprecipitation of human RIPK3 and human LCK in HEK293T cells. HEK293T cells were transfected with GST-LCK and/or Flag-RIPK3 expression constructs. Cells were harvested at 24h after transfection and cell lysates were immunoprecipitated with GST antibody (left) or RIPK3 antibody (right) (C). HEK293T cells were transfected with GST-LCK and/or Flag-RIPK3 expression constructs. Cells lysates were immunoprecipitated with RIPK3 antibody (left) or phosphor-Tyrosine antibody (right) (D).

Supporting information 6. Protein Interacting domains between RIPK3 and PP2A

A) Venn diagram represents the overlap of proteins and unique proteins identified by LC/MS/MS among TAP-purified samples as indicated. Total 503 proteins were identified as specific RIPK3 binding proteins.

B) Amino acid sequence of human RIPK3 (upper panel) and human PPP2R2A (lower panel).

C) In vitro kinase activity of RIPK3 toward PPP2R2A with ^{32}P-labeled ATP.

D) HEK293T cells were transfected with GFP-PPP2R2A and/or Flag-RIPK3 expression constructs. Cells were harvested at 24h after transfection.

Supporting information 7. Treatment of PP2A inhibitor or activator alters ERK phosphorylation status in thymic tumorigenesis.

A) Phosphorylation of ERK increased in response to PMA (1μM) and ionomycin (3μM) stimulation, which was further increased by LB-100 treatment.

B) Representative images of thymus from LB-100 or PBS injected mice (upper panel). LB-100 did not significantly change the thymus phenotype. Spleen, mesenteric lymph node and peripheral lymph node (lower) of each group were shown. (n=4 for each group)

C, D) Western blotting analysis of PPP2R2A and PPP2CA expression level in thymus tissues lysates from ENU-injected mice. The PP2A expression pattern was not affected by injection of LB-100 or SMAP.

E) Representative images of thymus from SMAP or PBS injected mice (upper panel). Injection of SMAP decreased thymus size in ENU-injected *Ripk3*$^{-/-}$ mice. Spleen, mesenteric lymph node and peripheral lymph node (lower) of each group were shown. (n=4 - 6 for each group)

Statistical analyses were performed using the two-tailed unpaired Student *t*-test. P values below 0.05 were considered significant in the following manner: *P<0.05, **P<0.01, ***P<0.001.

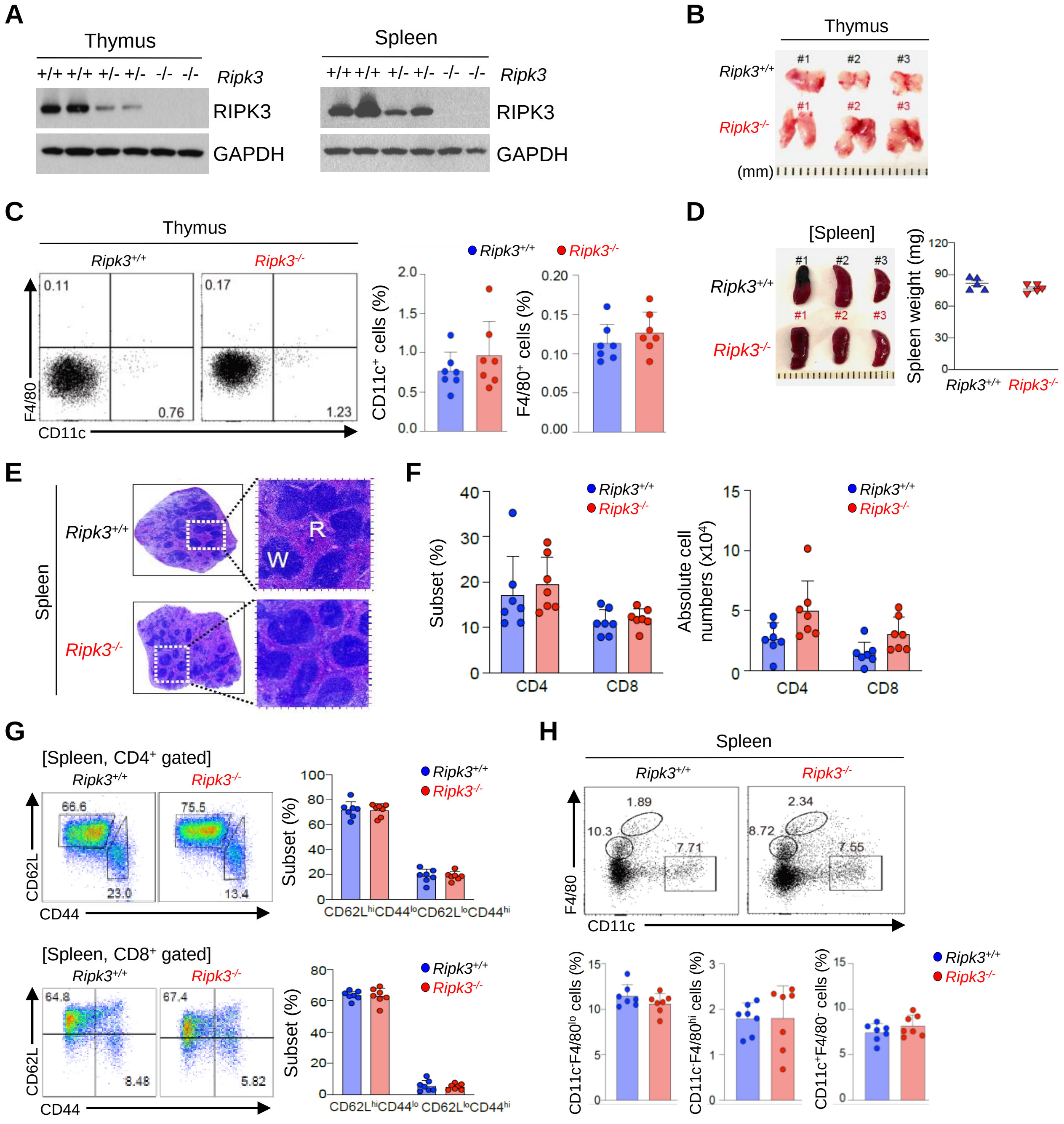

Supporting Information 1

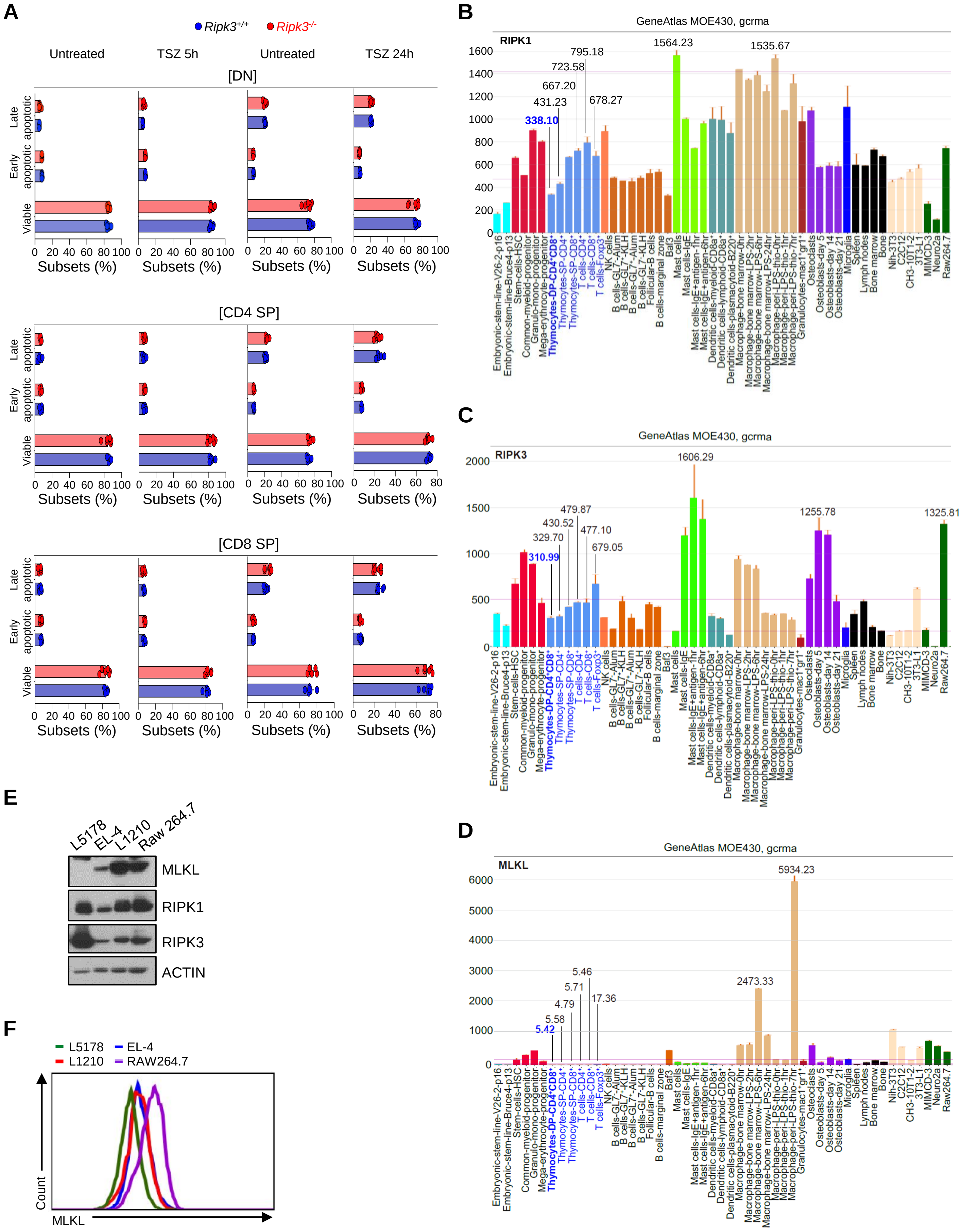

Supporting Information 2

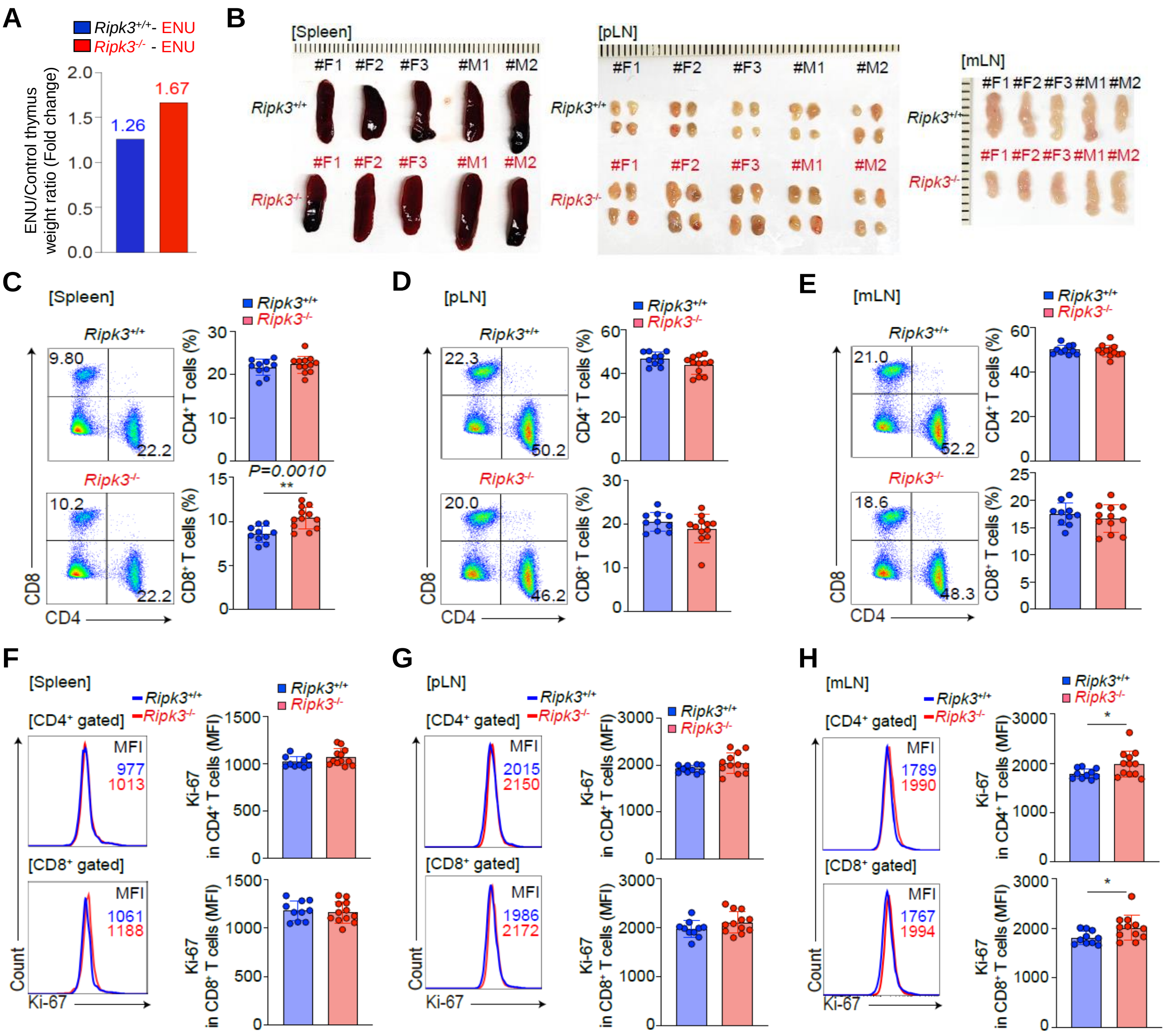

Supporting Information 3

A

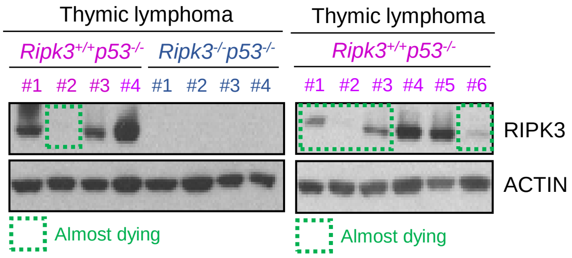

B

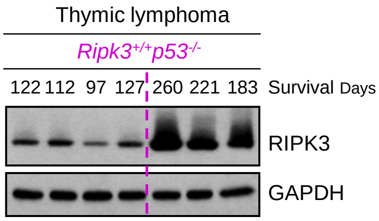

C

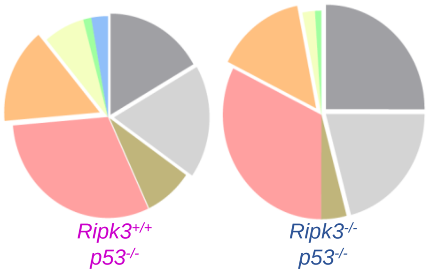

	Ripk3+/+ p53-/-	*Ripk3-/- p53-/-*
Thymic lymphoma	16.68 %	25.03 %
Thymic lymphoma (only)	18.06 %	20.86 %
Lymph node	8.34 %	4.17 %
Splenomegaly	30.57 %	32.29 %
Sarcoma	15.26 %	14.57 %
Kidney	6.92 %	2.06 %
Liver	1.37 %	1.03 %
Brain	2.80 %	
	100 %	100 %

Supporting Information 4

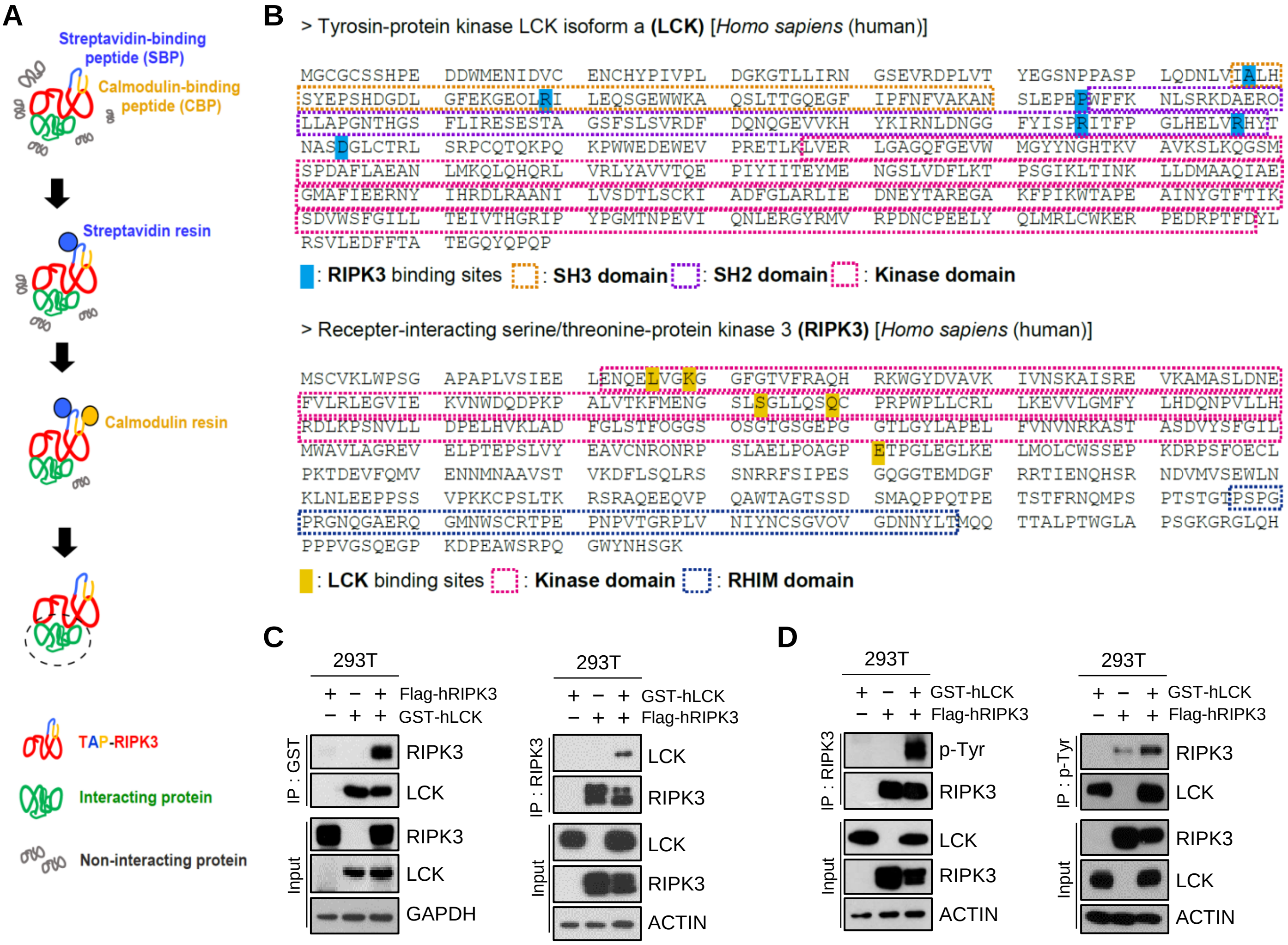

Supporting Information 5

A

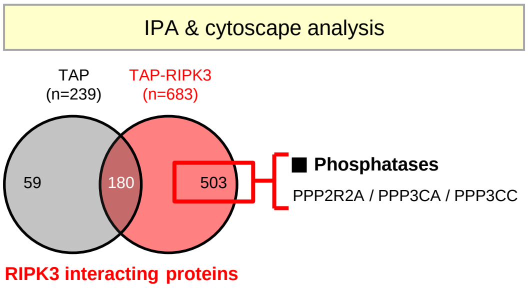

B

> Recepter-interacting serine/threonine-protein kinase 3 **(RIPK3)** [*Homo sapiens* (human)]

```
MSCVKLWPSG APAPLVSIEE LENQELVGKG GFGTVFRAQH RKWGYDVAVK IVNSKAISRE VKAMASLDNE
FVLRLEGVIE KVNWDQDPKP ALVTKFMENG SLSGLLQSQC PRPWPLLCRL LKEVVLGMFY LHDQNPVLLH
RDLKPSNVLL DPELHVKLAD FGLSTFQGGS QSGTGSGEPG GTLGYLAPEL FVNVNRKAST ASDVYSFGIL
MWAVLAGREV ELPTEPSLVY EAVCNRQNRP SLAELPQAGP ETPGLEGLKE LMQLCWSSEP KDRPSFQECL
PKTDEVFQMV ENNMNAAVST VKDFLSQLRS SNRRFSIPES GQGGTEMDGF RRTIENQHSR NDVMVSEWLN
KLNLEEPPSS VPKKCPSLTK RSRAQEEQVP QAWTAGTSSD SMAQPPQTPE TSTFRNQMPS PTSTGTPSPG
PRGNQGAERQ GMNWSCRTPE PNPVTGRPLV NIYNCSGVQV GDNNYLTMQQ TTALPTWGLA PSGKGRGLQH
PPPVGSQEGP KDPEAWSRPQ GWYNHSGK
```

: **PP2A-Bα551** binding sites : **Kinase domain** : **RHIM domain**

> Serine/threonine-protein phosphatase 2A 55kDa regulatory subunit B alpha isoform 1 **(PP2A-B55Bα)** [*Homo sapiens* (human)]

```
MAGAGGGNDI QWCFSQVKGA VDDDVAEADI ISTVEFNHSG ELLATGDKGG RVVIFQQEQE NKIQSHSRGE
YNVYSTFQSH EPEFDYLKSL EIEEKINKIR WLPQKNAAQF LLSTNDKTIK LWKISERDKR PEGYNLKEED
GRYRDPTTVT TLRVPVFRPM DLMVEASPRR IFANAHTYHI NSISINSDYE TYLSADDLRI NLWHLEITDR
SFNIVDIKPA NMEELTEVIT AAEFHPNSCN TFVYSSSKGT IRLCDMRASA LCDRHSKLFE EPEDPSNRSF
FSEIISSISD VKFSHSGRYM MTRDYLSVKI WDLNMENRPV ETYQVHEYLR SKLCSLYEND CIFDKFECCW
NGSDSVVMTG SYNNFFRMFD RNTKRDITLE ASRENNKPRT VLKPRKVCAS GKRKKDEISV DSLDFNKKIL
HTAWHPKENI IAVATTNNLY IFQDKVN
```

: **RIPK3** binding sites

C

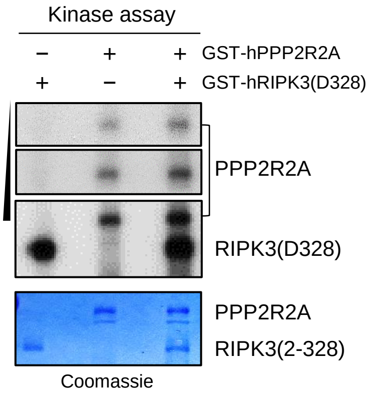

D

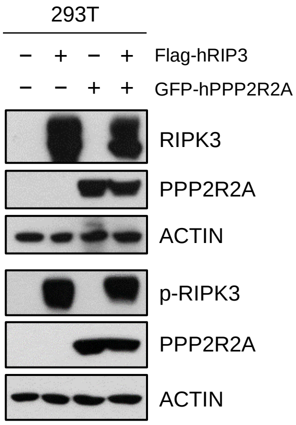

Supporting Information 6

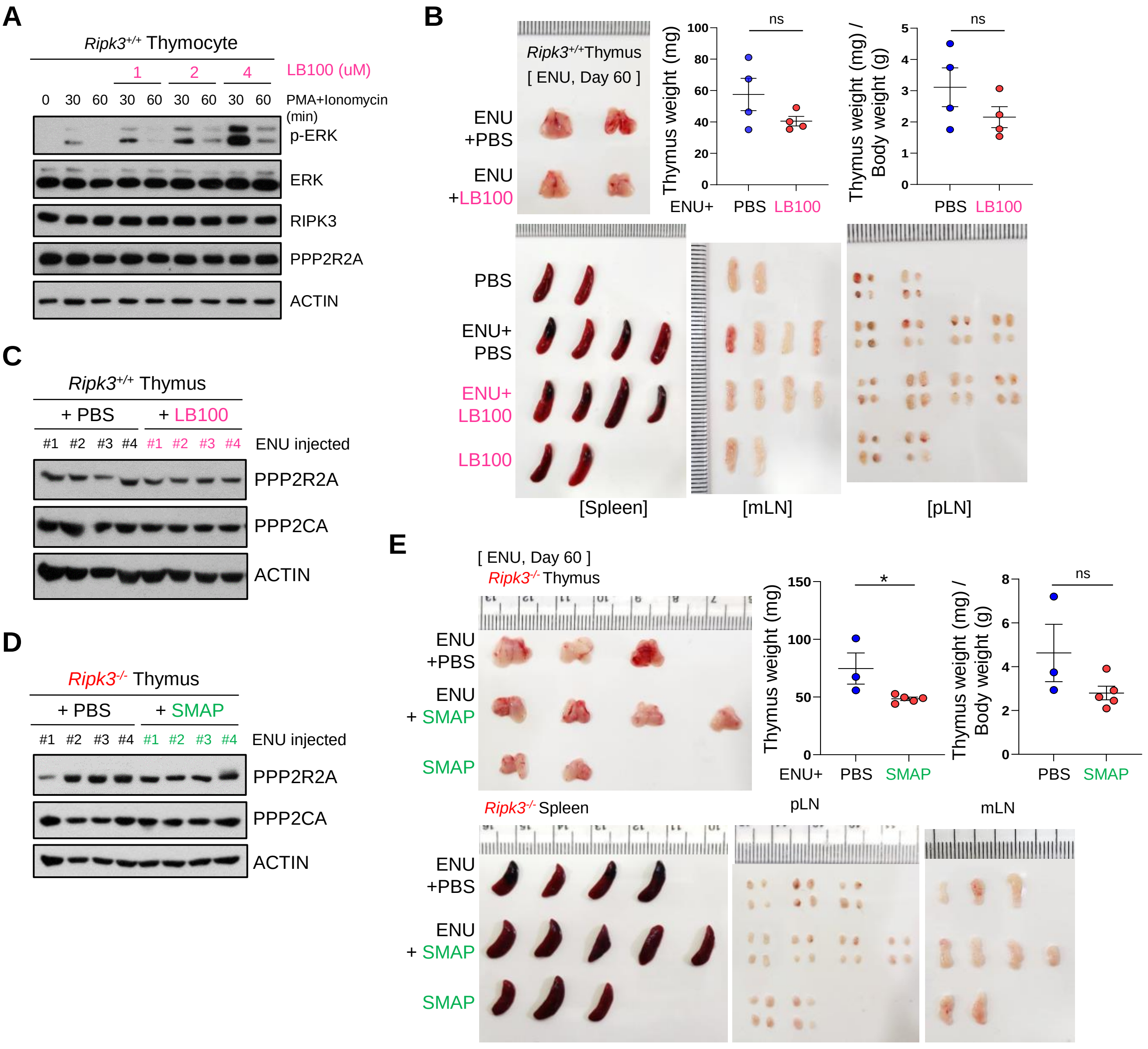

Supporting Information 7
